# Long non-coding RNA polymorphisms in 6p21.1 are associated with atrophic gastritis risk and gastric cancer prognosis

**DOI:** 10.18632/oncotarget.20115

**Published:** 2017-08-10

**Authors:** Zhi Lv, Liping Sun, Qian Xu, Yuehua Gong, Jingjing Jing, Chengzhong Xing, Yuan Yuan

**Affiliations:** ^1^ Tumor Etiology and Screening Department of Cancer Institute and General Surgery, The First Affiliated Hospital of China Medical University, Key Laboratory of Cancer Etiology and Prevention China Medical University, Liaoning Provincial Education Department, Shenyang 110001, China

**Keywords:** LncRNA, polymorphism, gastric cancer, susceptibility, prognosis

## Abstract

It has been suggested that the genetic variation in human chromosome 6p21.1 has potential importance for the susceptibility to gastric cancer (GC). The study aims to explore the relationship between the long non-coding RNA (lncRNA) polymorphisms in 6p21.1 and the risk of GC as well as atrophic gastritis (AG). Genotyping for eight single nucleotide polymorphisms (SNPs) was conducted using Sequenom MassARRAY platform in a total of 2507 northern Chinese subjects, including 749 GC cases, 878 AG cases and 880 controls. The results showed rs61516247 was associated with an increased AG risk in overall population (AA vs. GG: *P* = 0.046, OR = 1.46; A vs. G: *P* = 0.037, OR = 1.18). Four SNPs, rs61516247, rs1886753, rs7747696 and rs7749023 were associated with AG risk in some specific subgroups. Among them, rs1886753 had an interaction effect with *H.pylori* infection on AG risk (*P*_interaction_ = 0.038, OR = 1.62). In prognosis analysis, two SNPs, rs80112640 (AG+GG vs. AA: *P* = 0.047, HR = 0.56; G vs. A: *P* = 0.039, HR = 0.57) and rs72855279 (*P* = 0.043, HR = 0.57) were found to improve the overall survival of GC patients. In conclusion, lncRNA SNPs in 6p21.1 are associated with AG risk and GC prognosis. Our study provides all-new research clues for screening lncRNA-based biomarkers in the cancer-related hotspot region 6p21.1 with the potential to predict risk and prognosis of GC along with its precursor.

## INTRODUCTION

Genetic variation is a common phenomenon in the species evolution. As the most common form of genetic variation, the single nucleotide polymorphism (SNP) has been extensively investigated in the relationship with various diseases. SNPs can occur in different regions of chromosomes, changing structure and function of the genes involved.

Human chromosome 6 has more than 166 million base pairs. In 2003, the Welcome Trust Sanger researchers first reported that there were 2190 genes in chromosome 6 via sequencing analysis, of which 1557 were functional genes and about 130 were related to human diseases including hereditary hemochromatosis, Parkinson's disease, epilepsy, schizophrenia and heart disease etc. [1]. In 2010, the Genetic Epidemiology of Lung Cancer Consortium (GELCC) found that family lung cancer susceptibility gene was located in chromosome 6 by comparing the alleles of all 392 known genetic variants as genetic markers for both cancer patients and their healthy family members [2]. And in 2012, Guangfu Jin etc. conducted a large-scale case-control study by using combined samples of genome-wide association studies (GWAS) and replication stages, suggesting the potential importance of variants at 6p21.1 in the susceptibility to gastric cancer (GC) [3], which was the fourth common cancer worldwide and the second leading cause of cancer-related death [4]. Meanwhile, the association with GC of a polymorphism in the LRFN2 gene at that region was also revealed [3]. Subsequently, the SNPs in pepsinogen C (PGC), just located in 6p21.1, was found to play an important role in altering susceptibility to atrophic gastritis (AG) and GC by our research group in 2014 [5]. However, all the present studies focused on this hotspot region were related to protein-coding genes but few for non-coding RNAs (ncRNAs), with well-known significant gene regulative function. Long non-coding RNAs (lncRNAs) are 200-nt to 100-kb long, constituting the largest proportion of ncRNAs [6]. Accumulating studies have suggested lncRNAs are involved in the regulation of cell proliferation, invasion, metastasis and apoptosis in GC [7–9]. Currently, SNPs in six lncRNA genes have been reported to be associated with GC risk and prognosis, including H19, HOTAIR, TINCR, PRNCR1, NR_024015 and CASC8 [10–14]. However, it is remain unclear whether the lncRNA SNPs located in 6p21.1, the cancer-related hotspot region, are related to GC as well as its precancerous diseases.

In the present study, we conducted an analysis for the lncRNA SNPs at 6p21.1 in a northern Chinese population, aiming to explore their relationship with GC and AG. Our study might provide clues for screening novel biomarkers with the potential to predict risk and prognosis of GC along with its precursor.

## RESULTS

### Baseline characteristics of the subjects

The study subjects consisted of 878 AG, 749 GC, and two groups of gender- and age-matched controls, which were respectively 878 and 744 for AG and GC cases. *H.pylori* infection ratio was significantly higher in both AG and GC groups than control groups (*P* < 0.001). The proportion of individuals with drinking history in GC group was remarkably larger than the control group (*P* = 0.040). No significant difference in distribution of gender, age and smoking history was observed between any pairwise case and control groups (*P* > 0.05, Supplementary Table 1).

### Association of the studied SNPs with AG and GC risk

A total of eight SNPs were involved in the study based on our selection criteria. However, one of them entitled rs72854760 polymorphism was found not to be in accordance with HWE (*P* > 0.05), as a result of which, it was excluded from subsequent calculation. Reference frequencies of these SNPs in healthy controls (Beijing Han, China, NCBI database) were shown in Table 1.

**Table 1:** The association between the lncRNA SNPs and the risk of gastric diseases^a^

SNP genotypes	NCBI Ref	AG vs. CON				GC vs. CON			
		AG (%)	CON (%)	*P* (*P*_corr_)	OR (95%CI)	GC (%)	CON (%)	*P*	OR (95%CI)
**rs61516247**		*n* =874	*n* =876			*n* =749	*n* =742		
GG		402 (46.0)	437 (49.9)		1 (Ref)	357 (47.7)	364 (49.1)		1 (Ref)
GA		391 (44.7)	376 (42.9)	0.184	1.15 (0.94–1.41)	329 (43.9)	325 (43.8)	0.769	1.03 (0.83–1.29)
AA		81 (9.3)	63 (7.2)	**0.046 (0.322)**	**1.46 (1.01–2.12)**	63 (8.4)	53 (7.1)	0.371	1.20 (0.80–1.81)
GA+AA vs. GG				0.077	1.20 (0.98–1.46)			0.594	1.06 (0.86–1.31)
AA vs. GA+GG				0.089	1.37 (0.95–1.96)			0.362	1.20 (0.81–1.78)
A vs. G				**0.037 (0.259)**	**1.18 (1.01–1.37)**			0.430	1.07 (0.91–1.26)
*P*_HWE_	NA		0.141				0.088		
**rs1886753**	*n* =82	*n* =870	*n* =873			*n* =747	*n* =740		
AA	32 (39.0)	258 (29.7)	225 (25.8)		1 (Ref)	199 (26.6)	198 (26.8)		1 (Ref)
AG	38 (46.3)	419 (48.2)	446 (51.1)	0.171	0.85 (0.67–1.07)	387 (51.8)	375 (50.7)	0.513	1.09 (0.85–1.40)
GG	12 (14.6)	193 (22.2)	202 (23.1)	0.338	0.87 (0.66–1.16)	161 (21.6)	167 (22.6)	0.833	0.97 (0.71–1.31)
AG+GG vs. AA				0.164	0.86 (0.69–1.07)			0.700	1.05 (0.83–1.33)
GG vs. AG+AA				0.822	0.97 (0.77–1.23)			0.503	0.92 (0.71–1.18)
G vs. A				0.310	0.93 (0.81–1.07)			0.879	0.99 (0.85–1.15)
*P*_HWE_	0.895		0.507				0.677		
**rs80112640**		*n* =870	*n* =874			*n* =745	*n* =740		
AA		610 (70.1)	622 (71.2)		1 (Ref)	524 (70.3)	533 (72.0)		1 (Ref)
AG		235 (27.0)	225 (25.7)	0.448	1.09 (0.87–1.37)	205 (27.5)	185 (25.0)	0.422	1.10 (0.87–1.40)
GG		25 (2.9)	27 (3.1)	0.973	1.01 (0.56–1.82)	16 (2.1)	22 (3.0)	0.382	0.74 (0.38–1.45)
AG+GG vs. AA				0.474	1.08 (0.87–1.35)			0.591	1.07 (0.85–1.34)
GG vs. AG+AA				0.959	0.99 (0.55–1.76)			0.338	0.72 (0.37–1.41)
G vs. A				0.542	1.06 (0.88–1.28)			0.858	1.02 (0.83–1.25)
*P*_HWE_	NA		0.233				0.229		
**rs72855279**		*n* =875	*n* =873			*n* =748	*n* =739		
AA		614 (70.2)	624 (71.5)		1 (Ref)	528 (70.6)	535 (72.4)		1 (Ref)
AG		237 (27.1)	222 (25.4)	0.344	1.11 (0.89–1.40)	204 (27.3)	182 (24.6)	0.367	1.12 (0.88–1.42)
GG		24 (2.7)	27 (3.1)	0.952	0.98 (0.54–1.78)	16 (2.1)	22 (3.0)	0.378	0.74 (0.38–1.45)
AG+GG vs. AA				0.387	1.10 (0.89–1.37)			0.531	1.08 (0.85–1.36)
GG vs. AG+AA				0.870	0.95 (0.53–1.71)			0.331	0.72 (0.37–1.40)
G vs. A				0.481	1.07 (0.89–1.29)			0.801	1.03 (0.84–1.26)
*P*_HWE_	NA		0.187				0.180		
**rs7747696**		*n* =872	*n* =876			*n* =746	*n* =742		
AA		456 (52.3)	494 (56.4)		1 (Ref)	406 (54.4)	422 (56.9)		1 (Ref)
AG		350 (40.1)	313 (35.7)	0.081	1.20 (0.98–1.48)	296 (39.7)	264 (35.6)	0.383	1.10 (0.88–1.38)
GG		66 (7.6)	69 (7.9)	0.546	1.13 (0.77–1.65)	44 (5.9)	56 (7.5)	0.260	0.78 (0.51–1.20)
AG+GG vs. AA				0.086	1.19 (0.98–1.45)			0.670	1.05 (0.85–1.29)
GG vs. AG+AA				0.853	1.04 (0.72–1.50)			0.183	0.75 (0.49–1.15)
G vs. A				0.149	1.12 (0.96–1.32)			0.842	0.98 (0.83–1.17)
*P*_HWE_	NA		0.053				0.105		
**rs7748341**		*n* =872	*n* =872			*n* =748	*n* =738		
AA		592 (67.9)	602 (69.0)		1 (Ref)	510 (68.2)	515 (69.8)		1 (Ref)
AG		249 (28.6)	239 (27.4)	0.443	1.09 (0.87–1.36)	213 (28.5)	199 (27.0)	0.684	1.05 (0.83–1.33)
GG		31 (3.6)	31 (3.6)	0.778	1.08 (0.63–1.85)	25 (3.3)	24 (3.3)	0.874	1.05 (0.58–1.89)
AG+GG vs. AA				0.435	1.09 (0.88–1.35)			0.668	1.05 (0.84–1.32)
GG vs. AG+AA				0.860	1.05 (0.62–1.79)			0.904	1.04 (0.58–1.87)
G vs. A				0.462	1.07 (0.89–1.29)			0.680	1.04 (0.86–1.27)
*P*_HWE_	NA		0.233				0.379		
**rs7749023**	*n* =82	*n* =871	*n* =872			*n* =747	*n* =740		
AA	46 (56.1)	501 (57.5)	523 (60.0)		1 (Ref)	428 (57.3)	447 (60.4)		1 (Ref)
AC	28 (34.1)	317 (36.4)	292 (33.5)	0.209	1.15 (0.93–1.41)	281 (37.6)	246 (33.2)	0.293	1.13 (0.90–1.41)
CC	8 (9.8)	53 (6.1)	57 (6.5)	0.824	1.05 (0.69–1.59)	38 (5.1)	47 (6.4)	0.402	0.82 (0.52–1.30)
AC+CC vs. AA				0.240	1.13 (0.92–1.38)			0.482	1.08 (0.87–1.34)
CC vs. AC+AA				0.965	0.99 (0.66–1.49)			0.296	0.79 (0.50–1.24)
C vs. A				0.345	1.08 (0.92–1.28)			0.865	1.02 (0.85–1.21)
*P*_HWE_	0.238		0.065				0.099		

Note: ^a^, *P* was adjusted by gender, age and *H.pylori* infection status; NCBI Ref, reference frequencies of these SNPs in healthy controls (Beijing Han, China, NCBI database); AG, atrophic gastritis; GC, gastric cancer; CON, control; OR, odds ratio; CI, confidence interval; *P*_HWE_, Hardy-Weinberg Equilibrium in control groups; *P*_corr_, *P* values after Bonferroni correction. The results are in bold if *P* < 0.05.

First, the association between each SNP and gastric diseases risk in overall population was evaluated. Only rs61516247 polymorphism was found to be statistically significant, and both the homozygote variant AA and the allelic model were associated with an increased AG risk compared with the homozygote wild (AA vs. GG: *P* = 0.046, OR = 1.46, 95% CI = 1.01–2.12; A vs. G: *P* = 0.037, OR = 1.18, 95% CI = 1.01–1.37, Table 1).

We next divided GC into intestinal-type and diffused-type according to Lauren classification, estimating the association of the SNPs with each type of GC. However, no SNP demonstrated positive outcomes in any of genetic models (*P* > 0.05, Supplementary Table 2).

### Stratified analysis for the studied SNPs

To evaluate the association between the selected SNPs and gastric diseases risk in specific subgroups, we further performed stratified analyses based on the host characteristics. It was suggested four SNPs were associated with AG risk, including the rs61516247, rs1886753, rs7747696 and rs7749023 polymorphisms. For rs61516247, the homozygote variant AA, recessive model and allelic model could elevate AG risk significantly both in the subjects of age ≤ 60 years (*P* = 0.027, *P* = 0.049, *P* = 0.028, respectively) and non-smokers (*P* = 0.019, *P* = 0.028, *P* = 0.027, respectively). For rs1886753, all the genetic models other than recessive model were associated with a decreased AG risk in the *H.pylori*-positive subjects (AG vs. AA: *P* = 0.029; GG vs. AA: *P* = 0.030; dominant model: *P* = 0.016; G vs. A: *P* = 0.027); in the drinker group, its dominant model could also reduce AG risk (*P* = 0.048). For rs7747696, both the heterozygote AG and dominant model conferred an increased AG risk in the *H.pylori*-negative subjects (*P* = 0.043, *P* = 0.041, respectively); its G allele could elevate AG risk in the drinkers (*P* = 0.031). For rs7749023, individuals carried with the variant C allele had a 1.55-fold increased AG risk compared with the wild allele in the drinker group (*P* = 0.029, Supplementary Table 3).

### Haplotype analysis

Haplotype analyses were conducted to assess the association between haplotypes of these SNPs and gastric diseases risk. First, all the selected SNPs were included and seven haplotypes were found out. One of them was associated with a decreased AG risk (*P* = 0.017, OR = 0.83, 95% CI = 0.72–0.97). However, among the 7 SNPs, only four demonstrated significant associations with AG risk in previous analysis. To investigate whether the significance of the haplotype was contributed by the 4 SNPs, haplotype analysis for them was performed next, and one haplotype could reduce AG risk as well (*P* = 0.016, OR = 0.84, 95% CI = 0.72–0.97, Supplementary Table 4).

### Cumulative and interaction effects

**Figure 1:**
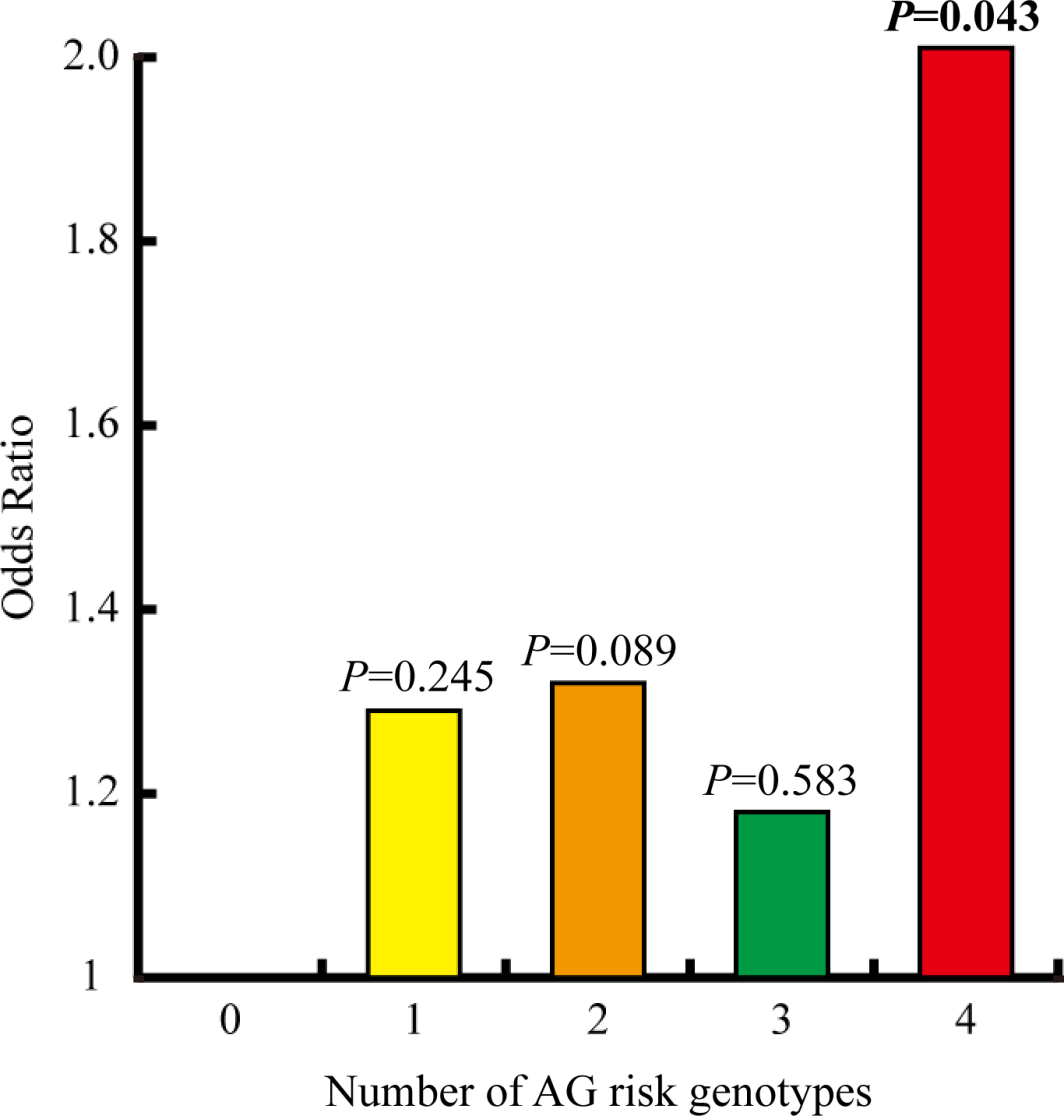
The cumulative effect of the four lncRNA SNPs associated with AG risk, including rs61516247, rs1886753, rs7747696 and rs7749023 The ORs vary from the number of risk genotypes the individuals carried with. A 2.01-fold increased AG risk could be obtained when the risk genotypes of the four SNPs were all combined for detection, which was the only group reaching significance (*P* = 0.043).

The contribution to gastric diseases risk when the selected SNPs were combined with each other was evaluated. Based on the results presented in Supplementary Table 3, we defined four genetic models as risk genotypes that elevate AG risk, which were AA for rs61516247, AG+GG for rs1886753, AG+GG for rs7747696 and CC for rs7749023. All the subjects were divided into four groups according to the number of risk genotypes they carried with, and individuals without any risk genotype were considered as control group (Figure 1). Other than the susceptibility to AG for individuals carried with four risk genotypes was remarkably increased when compared with the control group (*P* = 0.043, OR = 2.01, 95% CI = 1.02–3.97), no significant associations were shown in the other groups (*P* > 0.05).

The interactions between the SNPs and environmental factors were measured next. The wild genotype of rs1886753 was found to have a positive interaction effect with *H.pylori* infection on AG risk (*P*_interaction_ = 0.038, Table 2 and Supplementary Table 5). No interaction of three dimensions in AG risk was observed among the rs1886753 polymorphism and environmental factors (Supplementary Table 6).

**Table 2:** The interaction effects between the lncRNA SNPs and environmental factors on AG risk

SNP genotypes	H.pylori Infection^a^		Smoking^b^		Drinking^b^	
	Negative	Positive	No	Yes	No	Yes
**rs61516247**	*n* = 997	*n* = 753	*n* = 767	*n* = 378	*n* = 867	*n* =276
GG						
Case/Control	167/308	235/129	178/207	77/97	200/226	53/75
OR (95%CI)	1 (Ref)	3.36 (2.53–4.47)	1 (Ref)	0.92 (0.64–1.32)	1 (Ref)	0.80 (0.54–1.19)
GA+AA						
Case/Control	200/322	272/117	196/186	95/109	219/222	73/75
OR (95%CI)	1.15 (0.89–1.48)	4.29 (3.22–5.71)	1.23 (0.92–1.63)	1.01 (0.72–1.42)	1.12 (0.85–1.46)	1.10 (0.76–1.60)
	*P*_interaction_ = 0.591		*P*_interaction_ = 0.502		*P*_interaction_ = 0.887	
**rs1886753**	*n* = 988	*n* = 755	*n* = 766	*n* = 376	*n* = 865	*n* =275
AG+GG						
Case/Control	268/459	344/189	269/288	118/155	308/332	78/112
OR (95%CI)	1 (Ref)	3.12 (2.47–3.94)	1 (Ref)	0.82 (0.61–1.09)	1 (Ref)	0.75 (0.54–1.04)
AA						
Case/Control	94/167	164/58	106/103	53/50	112/113	47/38
OR (95%CI)	0.96 (0.72–1.29)	4.84 (3.46–6.77)	1.10 (0.80–1.51)	1.14 (0.75–1.73)	1.07 (0.79–1.45)	1.33 (0.85–2.10)
	*P*_interaction_ = 0.038 (0.266^c^), OR (95%CI) = 1.62 (1.03–2.56)		*P*_interaction_ = 0.630		*P*_interaction_ = 0.144	
**rs80112640**	*n* = 992	*n* = 752	*n* = 767	*n* = 378	*n* = 869	*n* =274
AA						
Case/Control	248/449	362/173	273/276	116/138	306/307	83/108
OR (95%CI)	1 (Ref)	3.79 (2.99–4.81)	1 (Ref)	0.85 (0.63–1.15)	1 (Ref)	0.77 (0.56–1.07)
AG+GG						
Case/Control	116/179	144/73	102/116	56/68	116/140	41/42
OR (95%CI)	1.17 (0.89–1.55)	3.57 (2.59–4.93)	0.89 (0.65–1.22)	0.83 (0.56–1.23)	0.83 (0.62–1.11)	0.98 (0.62–1.55)
	*P*_interaction_ = 0.330		*P*_interaction_ = 0.811		*P*_interaction_ = 0.166	
**rs72855279**	*n* = 995	*n* = 753	*n* = 766	*n* = 378	*n* = 867	*n* =275
AA						
Case/Control	250/250	364/174	274/277	117/138	307/308	84/108
OR (95%CI)	1 (Ref)	3.77 (2.97–4.78)	1 (Ref)	0.86 (0.64–1.15)	1 (Ref)	0.78 (0.56–1.08)
AG+GG						
Case/Control	117/178	144/71	101/114	56/67	114/138	42/41
OR (95%CI)	1.18 (0.89–1.57)	3.65 (2.64–5.05)	0.90 (0.65–1.23)	0.85 (0.57–1.25)	0.83 (0.62–1.11)	1.03 (0.65–1.63)
	*P*_interaction_ = 0.382		*P*_interaction_ = 0.800		*P*_interaction_ = 0.113	
**rs7747696**	*n* = 994	*n* = 754	*n* = 768	*n* = 377	*n* = 868	*n* =275
AA						
Case/Control	185/360	271/134	202/218	86/113	230/248	57/84
OR (95%CI)	1 (Ref)	3.94 (3.00–5.17)	1 (Ref)	0.82 (0.59–1.15)	1 (Ref)	0.73 (0.50–1.07)
AG+GG						
Case/Control	180/269	236/113	173/175	85/93	190/200	68/66
OR (95%CI)	1.30 (1.01–1.69)	4.06 (3.05–5.41)	1.07 (0.80–1.42)	0.99 (0.69–1.40)	1.02 (0.78–1.34)	1.11 (0.76–1.63)
	*P*_interaction_ = 0.249		*P*_interaction_ = 0.624		*P*_interaction_ = 0.072	
**rs7748341**	*n* = 991	*n* = 753	*n* = 768	*n* = 379	*n* = 870	*n* =275
AA						
Case/Control	241/433	351/169	263/269	113/135	297/301	78/104
OR (95%CI)	1 (Ref)	3.73 (2.93–4.75)	1 (Ref)	0.86 (0.63–1.16)	1 (Ref)	0.76 (0.54–1.06)
AG+GG						
Case/Control	124/193	156/77	112/124	60/71	125/147	47/46
OR (95%CI)	1.15 (0.88–1.52)	3.64 (2.66–4.99)	0.92 (0.68–1.26)	0.86 (0.59–1.27)	0.86 (0.65–1.15)	1.04 (0.67–1.60)
	*P*_interaction_ = 0.449		*P*_interaction_ = 0.765		*P*_interaction_ = 0.100	
**rs7749023**	*n* = 993	*n* = 750	*n* = 767	*n* = 379		
AA						
Case/Control	204/379	297/144	223/236	94/116	253/262	63/91
OR (95%CI)	1 (Ref)	3.83 (2.95–4.98)	1 (Ref)	0.86 (0.62–1.19)	1 (Ref)	0.72 (0.50–1.03)
AC+CC						
Case/Control	162/248	208/101	151/157	79/90	167/186	63/59
OR (95%CI)	1.21 (0.94–1.58)	3.83 (2.86–5.13)	1.02 (0.76–1.36)	0.93 (0.65–1.32)	0.93 (0.71–1.22)	1.11 (0.75–1.64)
	*P*_interaction_ = 0.331		*P*_interaction_ = 0.802		*P*_interaction_ = 0.051	

Note: ^a^, *P* for interaction was adjusted by gender and age; ^b^, *P* for interaction was adjusted by gender, age and *H.pylori* infection status; ^c^, *P* value after Bonferroni correction; AG, atrophic gastritis; GC, gastric cancer; CON, control; OR, odds ratio; CI, confidence interval. The results are in bold if *P* for interaction < 0.05.

### Association of the studied SNPs with GC prognosis

The association between the SNPs and five clinicopathological parameters was evaluated at first. The rs61516247 and rs1886753 polymorphisms were found to be associated with several parameters (*P* < 0.05, Supplementary Table 7).

We next made an assessment for the effects of host characteristics on OS for GC patients, including all the epidemiological and clinicopathological parameters. It was observed that OS was significantly affected by macroscopic type, TNM stage, lymphatic metastasis and depth of invasion (*P* = 0.043, *P* < 0.001, *P* < 0.001, *P* < 0.001, respectively, Table 3). Therefore, multivariate analysis was subsequently performed adjusted by these factors.

**Table 3:** The association between host characteristics and overall survival of GC patients

Factors	GC patients	Death	MST (M)	*P*
Total	*n* = 353	*n* = 103		
Gender				0.742
Male	251	75	58.9^a^	
Female	102	28	47.1^a^	
Age				0.576
≤ 60	201	57	61.9^a^	
> 60	152	46	53.3^a^	
*H.pylori* Infection				0.635
Positive	189	59	54.5^a^	
Negative	164	44	61.1^a^	
Smoking				0.776
Ever Smoker	112	31	37.2^a^	
Never Smoker	175	47	37.2^a^	
Drinking				0.328
Drinker	99	25	37.6^a^	
Nondrinker	188	53	36.8^a^	
Macroscopic type				**0.043**
Borrmann I-II	74	24	64.0^a^	
Borrmann III-IV	243	77	38.0	
Lauren classification				0.122
Intestinal-type	132	34	62.9^a^	
Diffuse-type	217	67	53.7^a^	
TNM stage				**< 0.001**
I–II	176	16	72.1^a^	
III–IV	177	87	28.0	
Lymphatic metastasis				**< 0.001**
Positive	214	89	37.0	
Negative	139	14	71.3^a^	
Depth of invasion				**< 0.001**
T1 + T2	83	3	48.4^a^	
T3 + T4	182	63	37.0	

Note: GC, gastric cancer; MST (M), median survival time (months); ^a^, mean survival time was provided when MST could not be calculated. The results are in bold if *P* < 0.05.

**Figure 2:**
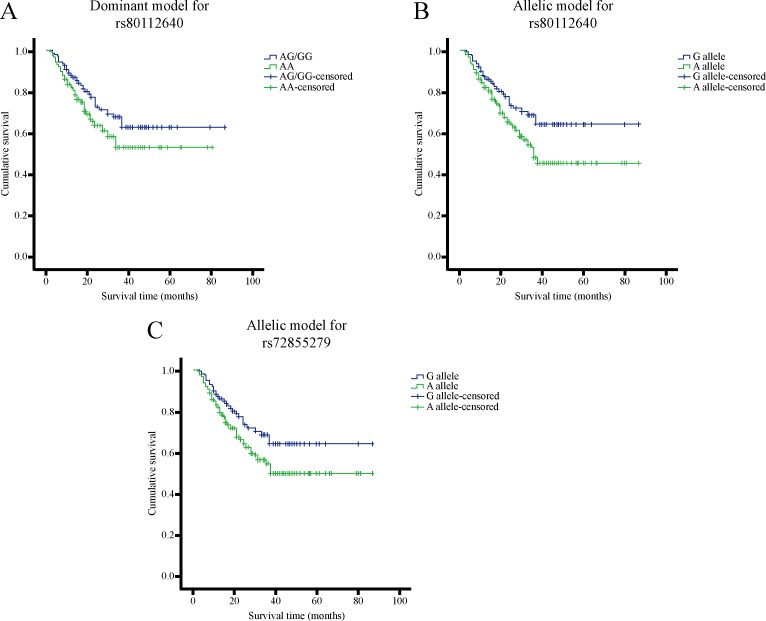
The survival curves for the genotypes of lncRNA SNPs with statistical significance in the overall survival of GC patients (**A**) rs80112640 AG+GG vs. AA; (**B**) rs80112640 G vs. A; (**C**) rs72855279 G vs. A.

Ultimately, the association between the SNPs and OS for GC patients was estimated both in univariate and multivariate analysis. The dominant model and the variant G allele of rs80112640 could improve GC prognosis in multivariate analysis (dominant model: *P* = 0.047, OR = 0.56, 95% CI = 0.31–0.99; G vs. A: *P* = 0.039, OR = 0.57, 95% CI = 0.33–0.97). Similar results were also shown in the allelic model of rs72855279 (*P* = 0.043, OR = 0.57, 95% CI = 0.33–0.98, Table 4). The corresponding survival curves were presented in Figure 2.

**Table 4:** The association between the lncRNA SNPs and prognosis of GC patients

SNP genotypes	GC patients	Death	MST (M)	Univariate		Multivariate	
				*P*	HR (95% CI)	*P* (*P*_corr_)	HR (95%CI)
**rs61516247**	*n* = 353	*n* =103					
GG	175	50	60.1^a^		1 (Ref)		1 (Ref)
GA	148	47	54.0^a^	0.935	1.02 (0.68–1.52)	0.767	0.93 (0.56–1.55)
AA	30	6	52.1^a^	0.286	0.63 (0.27–1.47)	0.489	0.65 (0.19–2.22)
GA+AA vs. GG				0.797	0.95 (0.65–1.40)	0.673	0.90 (0.54–1.48)
AA vs. GA+GG				0.248	0.62 (0.27–1.40)	0.348	0.57 (0.18–1.85)
A vs. G				0.477	0.90 (0.66–1.21)	0.476	0.87 (0.58–1.29)
**rs1886753**	*n* = 353	*n* =103					
AA	91	31	42.7^a^		1 (Ref)		1 (Ref)
AG	180	50	58.2^a^	0.294	0.79 (0.50–1.23)	0.900	0.96 (0.52–1.78)
GG	82	22	59.6^a^	0.337	0.77 (0.44–1.32)	0.288	0.68 (0.33–1.39)
AG+GG vs. AA				0.241	0.78 (0.51–1.19)	0.522	0.83 (0.46–1.48)
GG vs. AG+AA				0.665	0.90 (0.56–1.44)	0.251	0.72 (0.41–1.26)
G vs. A				0.328	0.87 (0.66–1.15)	0.258	0.82 (0.58–1.16)
**rs80112640**	*n* = 353	*n* =103					
AA	240	74	55.0^a^		1 (Ref)		1 (Ref)
AG	104	27	61.0^a^	0.415	0.83 (0.54–1.29)	0.082	0.60 (0.33–1.07)
GG	9	2	38.0^a^	0.505	0.62 (0.15–2.53)	0.968	NA
AG+GG vs. AA				0.342	0.81 (0.53–1.25)	**0.047 (0.329)**	**0.56 (0.31–0.99)**
GG vs. AG+AA				0.542	0.65 (0.16–2.62)	0.967	NA
G vs. A				0.309	0.82 (0.56–1.20)	**0.039 (0.273)**	**0.57 (0.33–0.97)**
**rs72855279**	*n* = 352	*n* =103					
AA	241	74	55.1^a^		1 (Ref)		1 (Ref)
AG	102	27	60.9^a^	0.445	0.84 (0.54–1.31)	0.092	0.61 (0.34–1.09)
GG	9	2	38.0^a^	0.507	0.62 (0.15–2.53)	0.968	NA
AG+GG vs. AA				0.367	0.82 (0.53–1.26)	0.052	0.56 (0.32–1.01)
GG vs. AG+AA				0.540	0.65 (0.16–2.62)	0.967	NA
G vs. A				0.329	0.83 (0.56–1.21)	**0.043 (0.301)**	**0.57 (0.33–0.98)**
**rs7747696**	*n* = 351	*n* =103					
AA	178	50	56.8^a^		1 (Ref)		1 (Ref)
AG	151	45	58.9^a^	0.872	1.03 (0.69–1.55)	0.420	0.81 (0.49–1.35)
GG	22	8	39.4^a^	0.638	1.20 (0.57–2.52)	0.623	0.77 (0.27–2.20)
AG+GG vs. AA				0.788	1.05 (0.72–1.55)	0.382	0.80 (0.49–1.31)
GG vs. AG+AA				0.650	1.18 (0.57–2.43)	0.781	0.87 (0.31–2.40)
G vs. A				0.698	1.06 (0.79–1.43)	0.444	0.86 (0.59–1.26)
**rs7748341**	*n* = 353	*n* =103					
AA	230	69	55.6^a^		1 (Ref)		1 (Ref)
AG	110	30	60.2^a^	0.691	0.92 (0.60–1.41)	0.228	0.71 (0.41–1.24)
GG	13	4	36.8^a^	0.739	0.84 (0.31–2.31)	0.490	0.60 (0.14–2.53)
AG+GG vs. AA				0.638	0.91 (0.60–1.37)	0.178	0.70 (0.41–1.18)
GG vs. AG+AA				0.754	0.85 (0.31–2.32)	0.546	0.65 (0.16–2.67)
G vs. A				0.607	0.91 (0.64–1.30)	0.181	0.73 (0.46–1.16)
**rs7749023**	*n* = 353	*n* =103					
AA	192	55	56.4^a^		1 (Ref)		1 (Ref)
AC	141	40	60.0^a^	0.944	0.99 (0.66–1.48)	0.247	0.74 (0.44–1.23)
CC	20	8	38.5^a^	0.548	1.26 (0.60–2.64)	0.612	0.76 (0.27–2.17)
AC+CC vs. AA				0.917	1.02 (0.69–1.50)	0.227	0.74 (0.45–1.21)
CC vs. AC+AA				0.523	1.27 (0.62–2.60)	0.816	0.89 (0.32–2.45)
C vs. A				0.740	1.05 (0.78–1.43)	0.305	0.81 (0.55–1.21)

Note: GC, gastric cancer; MST (M), median survival time (months); ^a^, mean survival time was provided when MST could not be calculated; HR, hazard ratio; CI, confidence interval; *P*_corr_, *P* values after Bonferroni correction. The results are in bold if *P* < 0.05.

## DISCUSSION

This case-control study explored the relationship of seven lncRNA SNPs in 6p21.1 with the risk and prognosis for GC and AG in a total of 2507 subjects. We newly found the rs61516247 polymorphism was associated with an increased AG risk in overall population. For the stratified analyses, associations with the susceptibility to AG were demonstrated in the rs61516247, rs1886753, rs7747696 and rs7749023 polymorphisms. Higher AG risk was observed when combining all these 4 SNPs. Very interestingly, the wild genotype of rs1886753 had a positive interaction effect with *H.pylori* infection, synergistically elevated AG risk. In addition, the rs80112640 and rs72855279 polymorphisms were found to improve OS for GC patients in multivariate analysis. To our knowledge, this is the first study about the relationship of lncRNA SNPs in the cancer-related hotspot region 6p21.1 with GC risk and prognosis, and it is also the first time to report the lncRNA SNPs associated with the susceptibility to AG.

It has been widely accepted that GC can develop from inflammation, atrophy, intestinal metaplasia and dysplasia. AG is considered as a precancerous condition of GC. To detect high-risk AG individuals could benefit the intervention and prevention of GC. In our study, three lncRNA genes at 6p21.1 were suggested to be associated with AG risk, including lnc-LRFN2-1, lnc-LRFN2-2 and lnc-C6orf132-1. As an important class of molecular regulators in human genomes, lncRNAs could result in various diseases by silencing or activating specific genes in epigenetic, transcriptional or posttranscriptional levels [15]. Based on the Database for Annotation, Visualization and Integrated Discovery (DAVID, https://david-d.ncifcrf.gov), we initially employed Gene Ontology (GO) analysis to obtain the function information of the three lncRNAs and their co-expressing genes from three aspects, including cell component (CC), biological process (BP) and molecular function (MF). Consequently, some lncRNAs were suggested to possibly contribute to AG initiation. For lnc-LRFN2-1, several co-expressing genes were found through Multi Experiment Matrix (MEM) [16]. GO analysis demonstrated they might target plasma membrane, concentrating on ion transport, channel activity and detection of external stimulus. Three co-expressing genes for lnc-LRFN2-2 were identified by our lncRNA expression profile, including CYP27B1, CACNA1I and GRIN2A, also shown to be associated with calcium ion transport in BP analysis. It has been reported that calcium ion could impair gastric mucosa through several pathways, leading to AG development [17]. Additionally, functional SNPs in lncRNA genes have been well accepted to exert regulatory roles in cancer [18, 19]. Therefore, it is reasonable to infer that the dysfunction of lnc-LRFN2-1 and lnc-LRFN2-2 caused by their SNPs might change the ion channel activity in membranes of gastric mucosal cells, making the epithelium more sensitive and vulnerable to environmental risk factors via calcium signaling pathway. However, all of the assumptions about the molecular mechanism need to be verified by further investigation.

Among the SNPs associated with AG risk, the rs61516247 polymorphism was statistically significant both in overall and stratified analysis. The risk effects demonstrated in its variant genotypes were more evident in younger subjects (age ≤ 60 years) and non-smokers. Tracing it to the cause, on the one hand, the defense of gastric mucosa to external hazards would become weakened as individuals grow old [20]; on the other hand, tobacco intake has been regarded as an independent risk factor for gastric diseases [21]. As a result, the association between rs61516247 and AG risk seems to be overlapped by aging and smoking. With respect to the rs1886753, rs7747696 and rs7749023 polymorphisms, they were all merely related to the subjects with or without *H.pylori* infection or drinking history, suggesting the association of the SNPs in overall subjects might be masked by *H.pylori* infection and alcohol consumption. From our perspective, it is also not difficult to figure out this phenomenon. Accumulated exposure to alcohol plays a crucial role in the progression of diseases [22]. Besides, *H.pylori* is one of the best-known environmental pathogenic factors, leading to gastric mucosa impaired after colonization in the stomach [23]. Interestingly, the variant genotypes of rs1886753 had protective effect on AG risk, while the wild AA was relatively a risk genotype, being able to elevate AG risk synergistically with *H.pylori* infection. Several studies have focused on the interaction between lncRNAs and *H.pylori*. Differentially expressed lncRNAs may play a partial or key role in the immune response to *H.pylori* [24]. And *H.pylori* infection might promote GC by deregulating lncRNAs expression [25]. However, further investigations are needed to elucidate whether the lncRNAs in 6p21.1 could interact with *H.pylori* and the specific mechanisms.

Due to the complex factors present in gastric diseases initiation, the capacity in recognition of susceptibility for one single polymorphism locus is limited [26, 27]. More advantages could be obtained when multiple SNPs are combined for detection. Our results showed the OR for AG risk calculated in the subjects carried with 4 risk genotypes simultaneously was almost doubled when compared with individuals carrying less risk genotypes, indicating a forceful cumulative effect of the SNPs. Obviously, better diagnostic efficacy for AG risk could be achieved when the rs61516247, rs1886753, rs7747696 and rs7749023 polymorphisms were all combined.

In the prognosis analysis, the rs80112640 and rs72855279 polymorphisms could both improve OS for GC patients after adjustments by several clinicopathological parameters. No significance was observed in univariate model, which was consistent with the results of analysis for OS-related factors. The two SNPs were located in the exon of lnc-C6orf132-1, of which the structural motifs might be affected and display a protective role for GC. However, the other SNPs in lnc-C6orf132-1, rs7747696 and rs7749023 were both associated with an increased AG risk, seemingly conflicting for the polymorphisms in the same lncRNA gene. Considering the results in function analysis of lnc-C6orf132-1, we believe this phenomenon could be explained to some extent. A number of co-expressing genes for lnc-C6orf132-1 were revealed in our lncRNA expression profile, shown to have bidirectional regulation effects on DNA transcription. That indicates lnc-C6orf132-1 has the ability to simultaneously upregulate and downregulate the expression of some relevant oncogenes or tumor suppressor genes when affected by different SNPs. As a result, the expression level of the same gene may vary from different stages during the progression of gastric diseases. Besides, the components associated with cancer outcome are quite complex, in which diverse factors might interact with each other. Therefore, it is comprehensible that the SNPs in lnc-C6orf132-1 cause contrary effects on AG risk and GC prognosis, while the specific mechanism still needs to be further investigated.

Several limitations should be acknowledged in our study. Firstly, the existence of data missing might influence the efficacy of statistical analysis to some extent, including SNP genotypes and epidemiological data. Secondly, the lncRNA SNPs in 6p21.1 region are not completely covered, which needs supplements in the future. Furthermore, our research is only focused on the association study without in-depth investigation about involved mechanisms. In the future functional studies need to be conducted to investigate the specific mechanism pathways in which the polymorphisms take effects.

In summary, we performed a case-control study to explore the relationship of the lncRNA SNPs in the cancer-related hotspot region 6p21.1 with the risk and prognosis for AG and GC in a Chinese population. Four SNPs were suggested to be associated with the susceptibility to AG in overall or stratified analysis, including the rs61516247, rs1886753, rs7747696 and rs7749023 polymorphisms. Two SNPs, rs80112640 and rs72855279 were found to be associated with OS for GC patients, of which the variant genotypes both indicated a better GC prognosis. These findings demonstrated the lncRNA polymorphisms in 6p21.1 might have the potential to become prediction biomarkers for AG risk and GC prognosis. The study would provide important clues for further research in this field, and also be guidance for the early diagnosis as well as individualized therapy of gastric diseases. Very interestingly, the lncRNA genes where our studied SNPs located are just adjacent to PGC, a specific marker related to gastric diseases quite intimately. Therefore, our study might also provide research clues for the exploration of the interaction effects between genetic variation of PGC and its neighbour lncRNA genes on the susceptibility to GC along with its precursor.

## MATERIALS AND METHODS

### Study participants

The study was approved by the Ethics Committee of the First Affiliated Hospital of China Medical University. Written informed consent was obtained from all participants. A total of 2507 subjects were involved in our study, including 749 GC, 878 AG and 880 controls. All enrolled individuals were recruited from the Zhuanghe Gastric Diseases Screening Program or hospitals in Zhuanghe and Shenyang of Liaoning Province, China between 2002 and 2013, which had been previously reported [28]. The controls were matched to the AG and GC cases on the basis of gender and age (± 5 years), respectively. After admission, gastroscopy examination was performed by experienced endoscopists. Four biopsy specimens were obtained from the gastric body, angulus, antrum and site of the lesion. Histopathological diagnoses were carried out independently by two gastrointestinal pathologists according to the updated Sydney system [29, 30]. Patients confirmed to have moderate to severe AG with or without intestinal metaplasia were selected for the AG group. And individuals in the control group were confirmed to be with normal stomach or to have mild superficial gastritis. Fasting venous blood samples (5ml) were collected from each subject.

### Information collection

Epidemiological data for each participant was obtained from medical records of inpatients or face-to-face inquiry. For the prognosis study, patients with GC who underwent surgical treatment were selected for regular follow-up after operation, which was completed by September 2014. Ultimately, a total of 353 GC cases with information of survival status and overall survival time were involved. Their clinicopathological data was obtained from the histopathological diagnoses. Clinical staging for GC was based on the seventh edition of UICC TNM staging [31]. Borrmann and Lauren typing were used for macroscopic and histological classification for GC, respectively.

### Determination of serum Helicobacter pylori (H.pylori)-IgG titer

The serum *H.pylori*-IgG titer was detected using an enzyme-linked immunosorbent assay (ELISA kit, Biohit, Helsinki, Finland). Individuals with serum *H.pylori*-IgG titer > 34IU were diagnosed as *H.pylori*-positive.

### SNP selection

We focused on the lncRNA genes located in human chromosome 6p21.1 region using Ensembl genome browser (http://asia.ensembl.org/index.html). Ranging from 40.00Mb to 43.00Mb in chromosome 6, a total of 3Mb sequences were encompassed from the origin of 6p21.1. First of all, functional SNPs were ought to be selected as far as possible. However, few current databases related to functional SNPs contained the information of lncRNAs in this region. As a result, selection was performed in terms of the location. Polymorphisms in the transcribed region are very likely to exert function via directly affecting gene expression [6], thus the SNPs in exon region of genes were taken into account. Subsequently, for the sake of our study practicability, eight SNPs in four lncRNA genes were selected on the basis of the following criteria: (1) minor allele frequency (MAF) > 0.05 in the CHB and JPT population; (2) pairwise linkage disequilibrium (r^2^ > 0.8). Among them, the rs1886753, rs61516247 and rs72854760 polymorphisms came from lnc-LRFN2-1, lnc-LRFN2-2 and lnc-LRFN2-3 respectively; the rs80112640, rs72855279, rs7747696, rs77483441 and rs7749023 polymorphisms all came from lnc-C6orf132-1.

### Genotyping

Genomic DNA was extracted from each blood sample using phenol-chloroform method. SNP genotyping was performed by Bio Miao Biological Technology (Beijing, China) applying Sequenom MassARRAY platform (Sequenom, San Diego, CA). Additionally, we randomly selected 10% of the samples for repeated assays and the results of all duplicated samples were 100% consistent.

### Statistical analysis

Hardy-Weinberg equilibrium (HWE) for each SNP in control groups was evaluated using the chi-square test. The χ^2^ test was applied to assess the differences in the epidemiological characteristics between case and control groups. The association between SNPs and gastric diseases risk as well as the clinicopathological parameters was estimated by calculating odds ratios (ORs) and their 95% confidence intervals (95%CIs) using multinomial logistic regression adjusted by gender, age and *H.pylori* infection status unless the *H.pylori* was regarded as a stratification item. The log likelihood ratio test was employed to evaluate the interactions among the SNPs and environmental factors. Kaplan-Meier method was used to calculate median survival time (MST); mean survival time was chosen when MST could not be calculated. Log rank test was used for evaluating the equality of survival distribution between different groups. The effects of SNPs on overall survival (OS) for GC patients were estimated by calculating hazard ratios (HRs) and their 95%CIs using cox regression both in univariate and multivariate models. The statistical analyses mentioned above were all conducted by using SPSS 22.0 software (SPSS, Chicago, IL). Haplotype analysis was performed by SHEsis online software (http://analysis.bio-x.cn/myAnalysis.php). All the tests were two-sided and *P* < 0.05 was considered to be statistically significant. The Bonferroni correction was used to adjust *P* values for multiple measures as needed. Additionally, the dominant and recessive genetic models were defined as heterozygote+homozygote variant vs. homozygote wild and homozygote variant vs. heterozygote+homozygote wild, respectively.

## SUPPLEMENTARY MATERIALS TABLES










