# Supplementary material for: Long non-coding RNA polymorphisms in 6p21.1 are associated with atrophic gastritis risk and gastric cancer prognosis
Source: Oncotarget. 2017 Aug 10;8(56):95303–15. doi: 10.18632/oncotarget.20115 (PMC5707023; doi:10.18632/oncotarget.20115)
Supplement: Supplementary file 1 [file oncotarget-08-95303-s001.pdf]

Long non-coding RNA polymorphisms in 6p21.1 are associated with atrophic gastritis risk and gastric cancer prognosis

SUPPLEMENTARY MATERIALS

Supplementary Table 1: The baseline characteristics of the subjects

Variables	AG vs. CON		GC vs. CON	
	AG (%)	CON (%)	GC (%)	CON (%)
Total	*n* = 878	*n* = 878	*n* = 749	*n* = 744
Gender	*P* = 0.962		*P* = 0.674	
Male	494 (56.3)	493 (56.2)	506 (67.6)	495 (66.5)
Female	384 (43.7)	385 (43.8)	243 (32.4)	249 (33.5)
Age	*P* = 0.170		*P* = 0.231	
Mean±SD	55.4 ± 9.7	54.8 ± 9.1	56.5 ± 9.8	55.9 ± 9.3
Median	56	54	57	56
Range	16–83	17–85	21–84	17–85
H.pylori Infection	***P* < 0.001**		***P* < 0.001**	
Positive	509 (58.0)	247 (28.1)	383 (51.1)	208 (28.0)
Negative	369 (42.0)	631 (71.9)	366 (48.9)	536 (72.0)
Smoking	*n* = 549	*n* = 601	*n* = 342	*n* = 515
	P = 0.291		*P* = 0.163	
Ever Smoker	173 (31.5)	207 (34.4)	153 (44.5)	204 (39.7)
Never Smoker	376 (68.5)	394 (65.6)	191 (55.5)	310 (60.3)
Drinking	*n* = 548	*n* = 606	*n* = 312	*n* = 519
	P = 0.390		***P* = 0.040**	
Drinker	126 (23.0)	151 (25.2)	111 (36.2)	150 (29.2)
Nondrinker	422 (77.0)	449 (74.8)	196 (63.8)	363 (70.8)

Note: AG, atrophic gastritis; GC, gastric cancer; CON, control. The results are in bold if *P* < 0.05.

Supplementary Table 2: The association between the lncRNA SNPs and GC risk of intestinal-type and diffused-type[a]. See Supplementary_Table_2

Supplementary Table 3: The association between the lncRNA SNPs and the risk of gastric diseases stratified by host characteristics. See Supplementary_Table_3

Supplementary Table 4: The association between haplotypes of the lncRNA SNPs and the risk of gastric diseases

	AG vs. CON				GC vs. CON			
**Haplotypes**	**AG (%)**	**CON (%)**	**P (Pcorr)**	**OR (95%CI)**	**GC (%)**	**CON (%)**	***P***	**OR (95%CI)**
7 SNPs[a]								
A-A-A-A-A-A-A	328.90 (19.3)	303.86 (17.7)	0.134	1.14 (0.96-1.36)	270.10 (18.3)	263.23 (18.1)	0.913	1.01 (0.84-1.22)
A-A-G-G-G-G-C	72.42 (4.2)	79.43 (4.6)	0.680	0.93 (0.67-1.29)	63.29 (4.3)	70.76 (4.9)	0.444	0.87 (0.62-1.24)
A-G-A-A-A-A-A	71.79 (4.2)	60.65 (3.5)	0.251	1.23 (0.87-1.74)	48.62 (3.3)	50.91 (3.5)	0.748	0.94 (0.63-1.40)
G-A-A-A-A-A-A	342.62 (20.1)	345.90 (20.1)	0.795	1.02 (0.86-1.21)	307.01 (20.8)	302.73 (20.8)	0.963	1.00 (0.83-1.19)
G-A-G-G-G-G-C	76.55 (4.5)	63.02 (3.7)	0.182	1.26 (0.90-1.77)	60.45 (4.1)	46.17 (3.2)	0.187	1.30 (0.88-1.92)
G-G-A-A-A-A-A	490.61 (28.7)	567.57 (33.0)	**0.017 (0.119)**	**0.83 (0.72-0.97)**	473.27 (32.0)	471.13 (32.3)	0.806	0.98 (0.84-1.15)
G-G-G-G-G-G-C	116.15 (6.8)	119.58 (7.0)	0.986	1.00 (0.77-1.30)	102.01 (6.9)	97.07 (6.7)	0.813	1.04 (0.78-1.38)
4 SNPs related to AG[b]								
A-A-A-A	331.67 (19.3)	305.83 (17.7)	0.167	1.13 (0.95-1.34)	269.30 (18.1)	262.82 (17.9)	0.952	1.01 (0.83-1.21)
A-A-G-C	117.96 (6.9)	110.43 (6.4)	0.514	1.09 (0.84-1.43)	112.68 (7.6)	96.22 (6.5)	0.301	1.16 (0.88-1.54)
A-G-A-A	71.89 (4.2)	60.65 (3.5)	0.272	1.22 (0.86-1.72)	48.46 (3.3)	50.84 (3.5)	0.731	0.93 (0.63-1.39)
G-A-A-A	340.13 (19.8)	347.14 (20.0)	0.964	1.00 (0.84-1.18)	309.92 (20.9)	304.11 (20.7)	0.991	1.00 (0.84-1.19)
C-A-G-C	108.05 (6.3)	106.74 (6.2)	0.823	1.03 (0.78-1.36)	76.67 (5.2)	86.94 (5.9)	0.339	0.86 (0.63-1.18)
G-G-A-A	496.13 (28.9)	570.33 (32.9)	**0.016 (0.112)**	**0.84 (0.72-0.97)**	476.30 (32.1)	476.20 (32.4)	0.712	0.97 (0.83-1.14)
G-G-G-C	175.44 (10.2)	168.84 (9.7)	0.585	1.06 (0.85-1.33)	151.13 (10.2)	139.15 (9.5)	0.572	1.07 (0.84-1.37)

Note: Haplotypes for [a], rs61516247-rs1886753-rs80112640-rs72855279-rs7747696-rs7748341-rs7749023; Haplotypes for [b], rs61516247-rs18867539-rs7747696-rs7749023; AG, atrophic gastritis; GC, gastric cancer; CON, control; OR, odds ratio; CI, confidence interval; P_{corr}, P values after Bonferroni correction. The results are in bold if $P < 0.05$.

Supplementary Table 5: The interaction effects between the lncRNA SNPs and environmental factors on GC risk. See Supplementary_Table_5

Supplementary Table 6: The interaction effects of three dimensions on AG risk among rs1886753 and environmental factors[a]

SNP genotypes	*H.pylori*	Smoking	Drinking	Case	Control	*P*	OR (95%CI)
				n = 546	*n* = 596		
AG+GG	(−)	(−)	/	105	215		1 (Ref)
AG+GG	(−)	(+)	/	50	112	0.665	0.91 (0.61–1.37)
AG+GG	(+)	(−)	/	164	73	< 0.001	4.60 (3.21–6.60)
AG+GG	(+)	(+)	/	68	43	< 0.001	3.24 (2.07–5.07)
AA	(−)	(−)	/	38	82	0.819	0.95 (0.61–1.49)
AA	(−)	(+)	/	17	36	0.916	0.97 (0.52–1.80)
AA	(+)	(−)	/	68	21	< 0.001	6.63 (3.86–11.40)
AA	(+)	(+)	/	36	14	< 0.001	5.27 (2.72–10.19)
						$P_{interaction}$= 0.971, OR (95%CI) = 1.02 (0.30–3.47)	
				n = 545	*n* = 595		
AG+GG	(−)	/	(−)	114	250		1 (Ref)
AG+GG	(−)	/	(+)	40	78	0.602	1.13 (0.72–1.75)
AG+GG	(+)	/	(−)	194	82	< 0.001	5.19 (3.69–7.29)
AG+GG	(+)	/	(+)	38	34	0.001	2.45 (1.47–4.09)
AA	(−)	/	(−)	37	88	0.720	0.92 (0.59–1.44)
AA	(−)	/	(+)	18	28	0.287	1.41 (0.75–2.65)
AA	(+)	/	(−)	75	25	< 0.001	6.58 (3.98–10.89)
AA	(+)	/	(+)	29	10	< 0.001	6.36 (3.00–13.49)
						$P_{interaction}$= 0.514, OR (95%CI) = 1.54 (0.42–5.69)	

Note: [a], *P* was adjusted by gender and age; OR, odds ratio; CI, confidence interval.

Supplementary Table 7: The association between the lncRNA SNPs and clinicopathological parameters[a]. See Supplementary_Table_7
