# Supplementary material for: Long non-coding RNA polymorphisms in 6p21.1 are associated with atrophic gastritis risk and gastric cancer prognosis
Source: Oncotarget. 2017 Aug 10;8(56):95303–15. doi: 10.18632/oncotarget.20115 (PMC5707023; doi:10.18632/oncotarget.20115)
Supplement: Supplementary file 2 [file oncotarget-08-95303-s002.docx]

| Table S2. The association between the lncRNA SNPs and GC risk of intestinal-type and diffused-type^a^ | | | | | | | |
| --- | --- | --- | --- | --- | --- | --- | --- |
| SNP genotypes | CON(%) | Intestinal-type GC(%) | Diffused-type GC(%) | Intestinal-type GC vs. CON | | Diffused-type GC vs. CON | |
|  |  |  |  | *P* | OR(95%CI) | *P* | OR(95%CI) |
| **rs61516247** | n=742 | n=215 | n=386 |  |  |  |  |
| GG | 364(49.1) | 103(47.9) | 179(46.4) |  | 1(Ref) |  | 1(Ref) |
| GA | 325(43.8) | 96(44.7) | 173(44.8) | 0.988 | 1.00(0.72-1.39) | 0.570 | 1.08(0.83-1.41) |
| AA | 53(7.1) | 16(7.4) | 34(8.8) | 0.899 | 1.04(0.56-1.95) | 0.238 | 1.34(0.83-2.17) |
| GA+AA vs. GG |  |  |  | 0.985 | 1.00(0.73-1.38) | 0.382 | 1.12(0.87-1.44) |
| AA vs. GA+GG |  |  |  | 0.914 | 1.03(0.56-1.90) | 0.242 | 1.32(0.83-2.10) |
| A vs. G |  |  |  | 0.954 | 1.01(0.79-1.29) | 0.247 | 1.12(0.92-1.36) |
| **rs1886753** | n=740 | n=214 | n=385 |  |  |  |  |
| AA | 198(26.8) | 54(25.2) | 103(26.8) |  | 1(Ref) |  | 1(Ref) |
| AG | 375(50.7) | 117(54.7) | 190(49.4) | 0.305 | 1.22(0.83-1.79) | 0.977 | 1.00(0.73-1.35) |
| GG | 167(22.6) | 43(20.1) | 92(23.9) | 0.870 | 0.96(0.60-1.55) | 0.646 | 1.09(0.76-1.56) |
| AG+GG vs. AA |  |  |  | 0.488 | 1.14(0.79-1.64) | 0.898 | 1.02(0.77-1.36) |
| GG vs. AG+AA |  |  |  | 0.378 | 0.84(0.57-1.24) | 0.565 | 1.09(0.81-1.47) |
| G vs. A |  |  |  | 0.934 | 0.99(0.79-1.24) | 0.675 | 1.04(0.87-1.24) |
| **rs80112640** | n=740 | n=214 | n=385 |  |  |  |  |
| AA | 533(72.0) | 140(65.4) | 273(70.9) |  | 1(Ref) |  | 1(Ref) |
| AG | 185(25.0) | 68(31.8) | 103(26.8) | 0.075 | 1.37(0.97-1.95) | 0.653 | 1.07(0.80-1.43) |
| GG | 22(3.0) | 6(2.8) | 9(2.3) | 0.959 | 0.98(0.38-2.54) | 0.565 | 0.79(0.35-1.77) |
| AG+GG vs. AA |  |  |  | 0.098 | 1.33(0.95-1.87) | 0.791 | 1.04(0.78-1.38) |
| GG vs. AG+AA |  |  |  | 0.789 | 0.88(0.34-2.29) | 0.534 | 0.78(0.35-1.73) |
| G vs. A |  |  |  | 0.174 | 1.23(0.91-1.64) | 0.974 | 1.00(0.78-1.29) |
| **rs72855279** | n=739 | n=215 | n=386 |  |  |  |  |
| AA | 535(72.4) | 141(65.6) | 273(70.7) |  | 1(Ref) |  | 1(Ref) |
| AG | 182(24.6) | 68(31.6) | 104(26.9) | 0.061 | 1.40(0.99-1.98) | 0.488 | 1.11(0.83-1.48) |
| GG | 22(3.0) | 6(2.8) | 9(2.3) | 0.958 | 0.98(0.38-2.54) | 0.571 | 0.79(0.35-1.78) |
| AG+GG vs. AA |  |  |  | 0.082 | 1.35(0.96-1.89) | 0.622 | 1.07(0.81-1.42) |
| GG vs. AG+AA |  |  |  | 0.785 | 0.88(0.34-2.28) | 0.524 | 0.77(0.35-1.72) |
| G vs. A |  |  |  | 0.152 | 1.24(0.92-1.66) | 0.819 | 1.03(0.80-1.32) |
| **rs7747696** | n=742 | n=212 | n=386 |  |  |  |  |
| AA | 422(56.9) | 107(50.5) | 208(53.9) |  | 1(Ref) |  | 1(Ref) |
| AG | 264(35.6) | 94(44.3) | 151(39.1) | 0.135 | 1.29(0.93-1.79) | 0.420 | 1.12(0.85-1.46) |
| GG | 56(7.5) | 11(5.2) | 27(7.0) | 0.346 | 0.71(0.35-1.44) | 0.916 | 0.97(0.59-1.61) |
| AG+GG vs. AA |  |  |  | 0.301 | 1.18(0.86-1.63) | 0.497 | 1.09(0.85-1.41) |
| GG vs. AG+AA |  |  |  | 0.197 | 0.63(0.32-1.27) | 0.759 | 0.93(0.57-1.51) |
| G vs. A |  |  |  | 0.770 | 1.04(0.81-1.34) | 0.678 | 1.04(0.85-1.28) |
| **rs7748341** | n=738 | n=214 | n=386 |  |  |  |  |
| AA | 515(69.8) | 136(63.6) | 263(68.1) |  | 1(Ref) |  | 1(Ref) |
| AG | 199(27.0) | 70(32.7) | 108(28.0) | 0.134 | 1.30(0.92-1.84) | 0.798 | 1.04(0.78-1.38) |
| GG | 24(3.3) | 8(3.7) | 15(3.9) | 0.704 | 1.18(0.50-2.76) | 0.545 | 1.23(0.63-2.43) |
| AG+GG vs. AA |  |  |  | 0.137 | 1.29(0.92-1.80) | 0.681 | 1.06(0.81-1.39) |
| GG vs. AG+AA |  |  |  | 0.867 | 1.08(0.46-2.53) | 0.561 | 1.22(0.62-2.40) |
| G vs. A |  |  |  | 0.180 | 1.22(0.91-1.62) | 0.579 | 1.07(0.84-1.35) |
| **rs7749023** | n=740 | n=214 | n=385 |  |  |  |  |
| AA | 447(60.4) | 113(52.8) | 222(57.7) |  | 1(Ref) |  | 1(Ref) |
| AC | 246(33.2) | 92(43.0) | 139(36.1) | 0.066 | 1.36(0.98-1.89) | 0.490 | 1.10(0.84-1.44) |
| CC | 47(6.4) | 9(4.2) | 24(6.2) | 0.360 | 0.70(0.33-1.50) | 0.924 | 1.03(0.60-1.75) |
| AC+CC vs. AA |  |  |  | 0.165 | 1.25(0.91-1.73) | 0.513 | 1.09(0.84-1.41) |
| CC vs. AC+AA |  |  |  | 0.191 | 0.60(0.28-1.29) | 0.965 | 0.99(0.59-1.67) |
| C vs. A |  |  |  | 0.534 | 1.09(0.84-1.41) | 0.605 | 1.06(0.86-1.31) |
| Note: ^a^, *P* was adjusted by gender, age and *H.pylori* infection status; CON, control; GC, gastric cancer; OR, odds ratio; CI, confidence interval. | | | | | | | |
