# Supplementary material for: Long non-coding RNA polymorphisms in 6p21.1 are associated with atrophic gastritis risk and gastric cancer prognosis
Source: Oncotarget. 2017 Aug 10;8(56):95303–15. doi: 10.18632/oncotarget.20115 (PMC5707023; doi:10.18632/oncotarget.20115)
Supplement: Supplementary file 3 [file oncotarget-08-95303-s003.docx]

| Table S3. The association between the lncRNA SNPs and the risk of gastric diseases stratified by host characteristics | | | | | | | |
| --- | --- | --- | --- | --- | --- | --- | --- |
| Variables | SNP genotypes | AG vs. CON | | | GC vs. CON | | |
|  |  | Case/Control | *P*(*P*_corr_) | OR(95%CI) | Case/Control | *P* | OR(95%CI) |
| **rs61516247** |  | 874/876 |  |  | 749/742 |  |  |
| Gender^a^ |  |  |  |  |  |  |  |
| Male | GG | 221/239 |  | 1(Ref) | 239/239 |  | 1(Ref) |
|  | GA | 227/220 | 0.607 | 1.08(0.82-1.42) | 216/221 | 0.865 | 0.98(0.75-1.28) |
|  | AA | 44/32 | 0.209 | 1.39(0.83-2.34) | 51/33 | 0.081 | 1.55(0.95-2.53) |
|  | GA+AA vs. GG |  | 0.410 | 1.12(0.86-1.46) |  | 0.704 | 1.05(0.81-1.36) |
|  | AA vs. GA+GG |  | 0.245 | 1.35(0.82-2.22) |  | 0.061 | 1.57(0.98-1.51) |
|  | A vs. G |  | 0.268 | 1.12(0.92-1.38) |  | 0.274 | 1.12(0.92-1.36) |
| Female | GG | 181/198 |  | 1(Ref) | 118/125 |  | 1(Ref) |
|  | GA | 164/156 | 0.178 | 1.24(0.91-1.69) | 113/104 | 0.440 | 1.16(0.80-1.69) |
|  | AA | 37/31 | 0.131 | 1.52(0.88-2.60) | 12/20 | 0.280 | 0.65(0.30-1.42) |
|  | GA+AA vs. GG |  | 0.097 | 1.29(0.96-1.73) |  | 0.680 | 1.08(0.75-1.56) |
|  | AA vs. GA+GG |  | 0.233 | 1.38(0.82-2.33) |  | 0.198 | 0.61(0.28-1.30) |
|  | A vs. G |  | 0.069 | 1.24(0.98-1.56) |  | 0.859 | 0.97(0.73-1.30) |
| Age^a^ |  |  |  |  |  |  |  |
| ≤60 | GG | 284/326 |  | 1(Ref) | 227/253 |  | 1(Ref) |
|  | GA | 270/270 | 0.224 | 1.16(0.91-1.48) | 213/219 | 0.583 | 1.08(0.83-1.41) |
|  | AA | 62/45 | **0.027(0.189)** | **1.63(1.06-2.50)** | 39/35 | 0.314 | 1.30(0.78-2.14) |
|  | GA+AA vs. GG |  | 0.081 | 1.23(0.98-1.55) |  | 0.436 | 1.11(0.86-1.43) |
|  | AA vs. GA+GG |  | **0.049(0.343)** | **1.52(1.00-2.31)** |  | 0.363 | 1.25(0.77-2.04) |
|  | A vs. G |  | **0.028(0.196)** | **1.22(1.02-1.46)** |  | 0.329 | 1.10(0.91-1.35) |
| >60 | GG | 118/111 |  | 1(Ref) | 130/111 |  | 1(Ref) |
|  | GA | 121/106 | 0.578 | 1.12(0.75-1.66) | 116/106 | 0.709 | 0.93(0.64-1.36) |
|  | AA | 19/18 | 0.738 | 1.14(0.54-2.42) | 24/18 | 0.858 | 1.07(0.53-2.15) |
|  | GA+AA vs. GG |  | 0.565 | 1.12(0.76-1.64) |  | 0.781 | 0.95(0.66-1.37) |
|  | AA vs. GA+GG |  | 0.918 | 1.04(0.51-2.14) |  | 0.759 | 1.11(0.57-2.17) |
|  | A vs. G |  | 0.630 | 1.07(0.80-1.44) |  | 0.933 | 1.00(0.75-1.31) |
| *H.pylori* Infection^a^ |  |  |  |  |  |  |  |
| Positive | GG | 235/129 |  | 1(Ref) | 167/308 |  | 1(Ref) |
|  | GA | 226/102 | 0.246 | 1.21(0.88-1.66) | 165/274 | 0.795 | 1.05(0.74-1.49) |
|  | AA | 46/15 | 0.116 | 1.65(0.88-3.08) | 35/48 | 0.477 | 1.29(0.64-2.59) |
|  | GA+AA vs. GG |  | 0.131 | 1.27(0.93-1.72) |  | 0.641 | 1.08(0.77-1.52) |
|  | AA vs. GA+GG |  | 0.172 | 1.52(0.83-2.79) |  | 0.444 | 1.30(0.66-2.55) |
|  | A vs. G |  | 0.081 | 1.24(0.97-1.58) |  | 0.501 | 1.10(0.84-1.43) |
| Negative | GG | 182/102 |  | 1(Ref) | 175/262 |  | 1(Ref) |
|  | GA | 169/92 | 0.462 | 1.11(0.85-1.45) | 160/233 | 0.841 | 1.03(0.78-1.36) |
|  | AA | 32/13 | 0.227 | 1.34(0.83-2.16) | 31/40 | 0.591 | 1.15(0.69-1.91) |
|  | GA+AA vs. GG |  | 0.315 | 1.14(0.88-1.48) |  | 0.736 | 1.05(0.80-1.37) |
|  | AA vs. GA+GG |  | 0.304 | 1.27(0.81-2.01) |  | 0.602 | 1.14(0.70-1.86) |
|  | A vs. G |  | 0.227 | 1.13(0.93-1.38) |  | 0.632 | 1.05(0.86-1.29) |
| Smoking^b^ |  |  |  |  |  |  |  |
| Ever Smoker | GG | 77/97 |  | 1(Ref) | 74/96 |  | 1(Ref) |
|  | GA | 82/94 | 0.751 | 1.08(0.69-1.68) | 68/92 | 0.950 | 0.99(0.62-1.58) |
|  | AA | 13/15 | 0.909 | 1.05(0.44-2.53) | 11/15 | 0.677 | 0.81(0.31-2.14) |
|  | GA+AA vs. GG |  | 0.740 | 1.08(0.70-1.66) |  | 0.888 | 0.97(0.61-1.53) |
|  | AA vs. GA+GG |  | 0.927 | 1.04(0.46-2.36) |  | 0.739 | 0.86(0.36-2.09) |
|  | A vs. G |  | 0.772 | 1.05(0.76-1.46) |  | 0.809 | 0.96(0.67-1.36) |
| Never Smoker | GG | 178/207 |  | 1(Ref) | 92/157 |  | 1(Ref) |
|  | GA | 157/162 | 0.300 | 1.19(0.86-1.64) | 84/133 | 0.725 | 1.07(0.72-1.59) |
|  | AA | 39/24 | **0.019(0.133)** | **2.02(1.13-3.63)** | 15/19 | 0.353 | 1.43(0.67-3.02) |
|  | GA+AA vs. GG |  | 0.102 | 1.29(0.95-1.76) |  | 0.552 | 1.12(0.77-1.64) |
|  | AA vs. GA+GG |  | **0.028(0.196)** | **1.89(1.07-3.35)** |  | 0.348 | 1.42(0.68-2.98) |
|  | A vs. G |  | **0.027(0.189)** | **1.31(1.03-1.67)** |  | 0.403 | 1.13(0.84-1.53) |
| Drinking^b^ |  |  |  |  |  |  |  |
| Drinker | GG | 53/75 |  | 1(Ref) | 57/74 |  | 1(Ref) |
|  | GA | 59/65 | 0.538 | 1.18(0.70-1.98) | 48/65 | 0.756 | 0.92(0.54-1.57) |
|  | AA | 14/10 | 0.186 | 1.86(0.74-4.69) | 6/10 | 0.412 | 0.62(0.19-1.97) |
|  | GA+AA vs. GG |  | 0.374 | 1.25(0.76-2.06) |  | 0.628 | 0.88(0.53-1.48) |
|  | AA vs. GA+GG |  | 0.267 | 1.64(0.68-3.94) |  | 0.482 | 0.67(0.22-2.03) |
|  | A vs. G |  | 0.248 | 1.25(0.86-1.81) |  | 0.517 | 0.87(0.58-1.31) |
| Nondrinker | GG | 200/226 |  | 1(Ref) | 93/176 |  | 1(Ref) |
|  | GA | 181/193 | 0.301 | 1.18(0.87-1.60) | 87/162 | 0.899 | 1.03(0.70-1.51) |
|  | AA | 38/29 | 0.059 | 1.73(0.98-3.07) | 16/24 | 0.398 | 1.37(0.66-2.81) |
|  | GA+AA vs. GG |  | 0.137 | 1.25(0.93-1.68) |  | 0.716 | 1.07(0.74-1.56) |
|  | AA vs. GA+GG |  | 0.094 | 1.60(0.92-2.76) |  | 0.348 | 1.41(0.69-2.86) |
|  | A vs. G |  | 0.064 | 1.24(0.99-1.56) |  | 0.514 | 1.10(0.83-1.47) |
| **rs1886753** |  | 870/873 |  |  | 747/740 |  |  |
| Gender^a^ |  |  |  |  |  |  |  |
| Male | AA | 157/132 |  | 1(Ref) | 145/133 |  | 1(Ref) |
|  | AG | 227/241 | 0.234 | 0.83(0.60-1.13) | 256/242 | 0.886 | 1.02(0.76-1.38) |
|  | GG | 107/116 | 0.366 | 0.84(0.58-1.22) | 104/116 | 0.273 | 0.81(0.56-1.18) |
|  | AG+GG vs. AA |  | 0.223 | 0.83(0.62-1.12) |  | 0.747 | 0.95(0.72-1.27) |
|  | GG vs. AG+AA |  | 0.770 | 0.95(0.70-1.31) |  | 0.170 | 0.81(0.59-1.10) |
|  | G vs. A |  | 0.336 | 0.91(0.76-1.10) |  | 0.312 | 0.91(0.76-1.09) |
| Female | AA | 101/93 |  | 1(Ref) | 54/65 |  | 1(Ref) |
|  | AG | 192/205 | 0.422 | 0.86(0.60-1.24) | 131/133 | 0.299 | 1.27(0.81-2.00) |
|  | GG | 86/86 | 0.683 | 0.91(0.59-1.41) | 57/51 | 0.179 | 1.47(0.84-2.58) |
|  | AG+GG vs. AA |  | 0.456 | 0.88(0.63-1.24) |  | 0.219 | 1.31(0.85-2.01) |
|  | GG vs. AG+AA |  | 0.987 | 1.00(0.70-1.43) |  | 0.413 | 1.20(0.77-1.87) |
|  | G vs. A |  | 0.654 | 0.95(0.77-1.18) |  | 0.221 | 1.18(0.91-1.52) |
| Age^a^ |  |  |  |  |  |  |  |
| ≤60 | AA | 185/162 |  | 1(Ref) | 125/135 |  | 1(Ref) |
|  | AG | 287/332 | 0.077 | 0.78(0.59-1.03) | 253/261 | 0.700 | 1.06(0.78-1.44) |
|  | GG | 140/146 | 0.568 | 0.91(0.65-1.26) | 99/111 | 0.913 | 0.98(0.67-1.43) |
|  | AG+GG vs. AA |  | 0.125 | 0.82(0.63-1.06) |  | 0.808 | 1.04(0.78-1.39) |
|  | GG vs. AG+AA |  | 0.687 | 1.06(0.80-1.39) |  | 0.687 | 0.94(0.69-1.28) |
|  | G vs. A |  | 0.465 | 0.94(0.80-1.11) |  | 0.935 | 0.99(0.83-1.19) |
| >60 | AA | 73/63 |  | 1(Ref) | 74/63 |  | 1(Ref) |
|  | AG | 132/114 | 0.938 | 1.02(0.65-1.60) | 134/114 | 0.450 | 1.19(0.76-1.85) |
|  | GG | 53/56 | 0.282 | 0.74(0.42-1.28) | 62/56 | 0.752 | 0.92(0.54-1.57) |
|  | AG+GG vs. AA |  | 0.705 | 0.92(0.60-1.41) |  | 0.711 | 1.08(0.71-1.64) |
|  | GG vs. AG+AA |  | 0.194 | 0.74(0.46-1.17) |  | 0.446 | 0.84(0.55-1.30) |
|  | G vs. A |  | 0.316 | 0.87(0.66-1.14) |  | 0.824 | 0.97(0.75-1.26) |
| *H.pylori* Infection^a^ |  |  |  |  |  |  |  |
| Positive | AA | 164/58 |  | 1(Ref) | 94/167 |  | 1(Ref) |
|  | AG | 239/129 | **0.029(0.203)** | **0.66(0.46-0.96)** | 180/317 | 0.296 | 0.81(0.54-1.21) |
|  | GG | 105/60 | **0.030(0.210)** | **0.62(0.40-1.00)** | 88/142 | 0.554 | 0.86(0.53-1.41) |
|  | AG+GG vs. AA |  | **0.016(0.112)** | **0.65(0.46-0.92)** |  | 0.346 | 0.83(0.57-1.22) |
|  | GG vs. AG+AA |  | 0.265 | 0.81(0.57-1.17) |  | 0.984 | 1.00(0.66-1.49) |
|  | G vs. A |  | **0.027(0.189)** | **0.78(0.63-0.97)** |  | 0.542 | 0.93(0.73-1.18) |
| Negative | AA | 113/53 |  | 1(Ref) | 86/145 |  | 1(Ref) |
|  | AG | 181/108 | 0.917 | 1.02(0.74-1.39) | 206/267 | 0.090 | 1.32(0.96-1.83) |
|  | GG | 88/47 | 0.612 | 1.10(0.76-1.59) | 73/120 | 0.860 | 1.04(0.70-1.54) |
|  | AG+GG vs. AA |  | 0.776 | 1.04(0.78-1.40) |  | 0.196 | 1.23(0.90-1.67) |
|  | GG vs. AG+AA |  | 0.522 | 1.11(0.81-1.50) |  | 0.368 | 0.86(0.62-1.19) |
|  | G vs. A |  | 0.576 | 1.05(0.88-1.27) |  | 0.781 | 1.03(0.85-1.24) |
| Smoking^b^ |  |  |  |  |  |  |  |
| Ever Smoker | AA | 53/50 |  | 1(Ref) | 40/50 |  | 1(Ref) |
|  | AG | 78/108 | 0.187 | 0.70(0.42-1.19) | 84/105 | 0.591 | 1.16(0.67-2.00) |
|  | GG | 40/47 | 0.876 | 0.95(0.52-1.75) | 28/47 | 0.547 | 0.80(0.39-1.65) |
|  | AG+GG vs. AA |  | 0.300 | 0.78(0.48-1.26) |  | 0.883 | 1.04(0.62-1.75) |
|  | GG vs. AG+AA |  | 0.512 | 1.19(0.71-1.98) |  | 0.190 | 0.69(0.39-1.21) |
|  | G vs. A |  | 0.791 | 0.96(0.71-1.30) |  | 0.504 | 0.90(0.65-1.24) |
| Never Smoker | AA | 106/103 |  | 1(Ref) | 53/89 |  | 1(Ref) |
|  | AG | 187/194 | 0.706 | 0.93(0.65-1.34) | 94/144 | 0.670 | 1.10(0.70-1.74) |
|  | GG | 82/94 | 0.422 | 0.83(0.53-1.30) | 44/75 | 0.753 | 1.09(0.64-1.86) |
|  | AG+GG vs. AA |  | 0.562 | 0.90(0.64-1.28) |  | 0.669 | 1.10(0.72-1.68) |
|  | GG vs. AG+AA |  | 0.638 | 0.92(0.64-1.32) |  | 0.952 | 1.01(0.65-1.59) |
|  | G vs. A |  | 0.519 | 0.93(0.75-1.16) |  | 0.758 | 1.04(0.80-1.37) |
| Drinking^b^ |  |  |  |  |  |  |  |
| Drinker | AA | 47/38 |  | 1(Ref) | 24/38 |  | 1(Ref) |
|  | AG | 54/75 | 0.059 | 0.57(0.32-1.02) | 62/74 | 0.509 | 1.24(0.65-2.36) |
|  | GG | 24/37 | 0.157 | 0.61(0.30-1.21) | 24/37 | 0.877 | 1.06(0.49-2.28) |
|  | AG+GG vs. AA |  | **0.048(0.336)** | **0.59(0.34-1.00)** |  | 0.618 | 1.17(0.64-2.14) |
|  | GG vs. AG+AA |  | 0.573 | 0.84(0.46-1.53) |  | 0.659 | 0.87(0.47-1.61) |
|  | G vs. A |  | 0.103 | 0.75(0.53-1.06) |  | 0.971 | 1.01(0.70-1.45) |
| Nondrinker | AA | 112/113 |  | 1(Ref) | 58/99 |  | 1(Ref) |
|  | AG | 211/228 | 0.769 | 0.95(0.67-1.35) | 94/176 | 0.924 | 1.02(0.66-1.59) |
|  | GG | 97/104 | 0.830 | 0.95(0.62-1.46) | 44/85 | 0.987 | 1.00(0.59-1.71) |
|  | AG+GG vs. AA |  | 0.777 | 0.95(0.68-1.33) |  | 0.994 | 1.00(0.66-1.52) |
|  | GG vs. AG+AA |  | 0.924 | 1.02(0.72-1.44) |  | 0.909 | 0.97(0.63-1.52) |
|  | G vs. A |  | 0.906 | 0.99(0.80-1.21) |  | 0.949 | 0.99(0.76-1.29) |
| **rs80112640** |  | 870/874 |  |  | 745/740 |  |  |
| Gender^a^ |  |  |  |  |  |  |  |
| Male | AA | 346/350 |  | 1(Ref) | 349/251 |  | 1(Ref) |
|  | AG | 130/126 | 0.507 | 1.11(0.82-1.50) | 143/127 | 0.403 | 1.13(0.85-1.51) |
|  | GG | 13/15 | 0.723 | 0.86(0.39-1.94) | 11/15 | 0.452 | 0.73(0.32-1.65) |
|  | AG+GG vs. AA |  | 0.601 | 1.08(0.81-1.45) |  | 0.548 | 1.09(0.82-1.44) |
|  | GG vs. AG+AA |  | 0.673 | 0.84(0.38-1.87) |  | 0.399 | 0.71(0.31-1.59) |
|  | G vs. A |  | 0.747 | 1.04(0.81-1.35) |  | 0.788 | 1.03(0.81-1.32) |
| Female | AA | 264/272 |  | 1(Ref) | 175/182 |  | 1(Ref) |
|  | AG | 105/99 | 0.683 | 1.07(0.77-1.50) | 62/58 | 0.839 | 1.05(0.68-1.60) |
|  | GG | 12/12 | 0.699 | 1.18(0.50-2.78) | 5/7 | 0.577 | 0.71(0.21-2.37) |
|  | AG+GG vs. AA |  | 0.623 | 1.09(0.79-1.50) |  | 0.962 | 1.01(0.67-1.53) |
|  | GG vs. AG+AA |  | 0.740 | 1.15(0.49-2.70) |  | 0.575 | 0.71(0.21-2.36) |
|  | G vs. A |  | 0.590 | 1.08(0.82-1.43) |  | 0.895 | 0.98(0.68-1.41) |
| Age^a^ |  |  |  |  |  |  |  |
| ≤60 | AA | 429/451 |  | 1(Ref) | 337/362 |  | 1(Ref) |
|  | AG | 170/169 | 0.636 | 1.07(0.82-1.38) | 128/129 | 0.832 | 1.03(0.77-1.38) |
|  | GG | 16/21 | 0.965 | 0.99(0.49-1.96) | 11/16 | 0.589 | 0.80(0.36-1.78) |
|  | AG+GG vs. AA |  | 0.673 | 1.06(0.82-1.36) |  | 0.952 | 1.01(0.76-1.34) |
|  | GG vs. AG+AA |  | 0.911 | 0.96(0.49-1.91) |  | 0.578 | 0.80(0.36-1.77) |
|  | G vs. A |  | 0.740 | 1.04(0.83-1.29) |  | 0.902 | 0.98(0.77-1.26) |
| >60 | AA | 181/171 |  | 1(Ref) | 187/171 |  | 1(Ref) |
|  | AG | 65/56 | 0.510 | 1.16(0.74-1.82) | 77/56 | 0.308 | 1.25(0.82-1.90) |
|  | GG | 9/6 | 0.883 | 0.92(0.29-2.88) | 5/6 | 0.360 | 0.55(0.16-1.97) |
|  | AG+GG vs. AA |  | 0.571 | 1.13(0.74-1.74) |  | 0.456 | 1.17(0.78-1.76) |
|  | GG vs. AG+AA |  | 0.825 | 0.88(0.28-2.74) |  | 0.297 | 0.51(0.14-1.81) |
|  | G vs. A |  | 0.670 | 1.09(0.75-1.58) |  | 0.716 | 1.07(0.75-1.54) |
| *H.pylori* Infection^a^ |  |  |  |  |  |  |  |
| Positive | AA | 362/173 |  | 1(Ref) | 263/147 |  | 1(Ref) |
|  | AG | 133/64 | 0.929 | 1.02(0.72-1.44) | 110/53 | 0.395 | 1.18(0.80-1.74) |
|  | GG | 11/9 | 0.195 | 0.55(0.22-1.36) | 7/7 | 0.245 | 0.53(0.18-1.55) |
|  | AG+GG vs. AA |  | 0.802 | 0.96(0.68-1.34) |  | 0.608 | 1.10(0.76-1.60) |
|  | GG vs. AG+AA |  | 0.189 | 0.55(0.22-1.35) |  | 0.196 | 0.50(0.17-1.44) |
|  | G vs. A |  | 0.528 | 0.91(0.68-1.22) |  | 0.944 | 1.01(0.73-1.40) |
| Negative | AA | 248/449 |  | 1(Ref) | 261/386 |  | 1(Ref) |
|  | AG | 102/161 | 0.342 | 1.15(0.86-1.55) | 95/132 | 0.711 | 1.06(0.78-1.44) |
|  | GG | 14/18 | 0.314 | 1.45(0.71-3.00) | 9/15 | 0.787 | 0.89(0.38-2.07) |
|  | AG+GG vs. AA |  | 0.245 | 1.18(0.89-1.56) |  | 0.783 | 1.04(0.78-1.40) |
|  | GG vs. AG+AA |  | 0.375 | 1.38(0.68-2.81) |  | 0.761 | 0.88(0.38-2.03) |
|  | G vs. A |  | 0.189 | 1.18(0.92-1.50) |  | 0.884 | 1.02(0.79-1.32) |
| Smoking^b^ |  |  |  |  |  |  |  |
| Ever Smoker | AA | 116/138 |  | 1(Ref) | 273/276 |  | 1(Ref) |
|  | AG | 51/64 | 0.860 | 0.96(0.60-1.54) | 93/101 | 0.132 | 0.67(0.40-1.13) |
|  | GG | 5/4 | 0.731 | 1.28(0.31-5.31) | 9/15 | 0.524 | 0.56(0.10-3.32) |
|  | AG+GG vs. AA |  | 0.929 | 0.98(0.62-1.55) |  | 0.112 | 0.66(0.40-1.10) |
|  | GG vs. AG+AA |  | 0.722 | 1.29(0.31-5.32) |  | 0.558 | 0.59(0.10-3.51) |
|  | G vs. A |  | 0.981 | 1.01(0.67-1.50) |  | 0.119 | 0.70(0.44-1.10) |
| Never Smoker | AA | 112/137 |  | 1(Ref) | 130/224 |  | 1(Ref) |
|  | AG | 37/62 | 0.604 | 0.91(0.64-1.30) | 55/73 | 0.461 | 1.18(0.76-1.82) |
|  | GG | 2/4 | 0.981 | 0.99(0.40-2.46) | 4/11 | 0.381 | 0.58(0.17-1.98) |
|  | AG+GG vs. AA |  | 0.596 | 0.91(0.65-1.28) |  | 0.656 | 1.10(0.72-1.68) |
|  | GG vs. AG+AA |  | 0.926 | 0.96(0.39-2.33) |  | 0.324 | 0.54(0.16-1.83) |
|  | G vs. A |  | 0.621 | 0.93(0.69-1.25) |  | 0.953 | 1.01(0.70-1.46) |
| Drinking^b^ |  |  |  |  |  |  |  |
| Drinker | AA | 83/108 |  | 1(Ref) | 75/108 |  | 1(Ref) |
|  | AG | 35/38 | 0.474 | 1.23(0.70-2.17) | 32/37 | 0.777 | 1.09(0.61-1.96) |
|  | GG | 6/4 | 0.302 | 2.08(0.52-8.32) | 2/4 | 0.566 | 0.59(0.10-3.62) |
|  | AG+GG vs. AA |  | 0.337 | 1.30(0.76-2.23) |  | 0.885 | 1.04(0.59-1.84) |
|  | GG vs. AG+AA |  | 0.342 | 1.91(0.50-7.27) |  | 0.554 | 0.58(0.10-3.50) |
|  | G vs. A |  | 0.244 | 1.32(0.83-2.09) |  | 0.962 | 0.99(0.60-1.63) |
| Nondrinker | AA | 306/307 |  | 1(Ref) | 139/254 |  | 1(Ref) |
|  | AG | 108/125 | 0.286 | 0.83(0.60-1.16) | 51/96 | 0.775 | 0.94(0.61-1.44) |
|  | GG | 8/15 | 0.584 | 0.77(0.30-1.98) | 4/11 | 0.477 | 0.64(0.18-2.21) |
|  | AG+GG vs. AA |  | 0.253 | 0.83(0.60-1.14) |  | 0.660 | 0.91(0.60-1.38) |
|  | GG vs. AG+AA |  | 0.632 | 0.80(0.31-2.02) |  | 0.495 | 0.65(0.19-2.24) |
|  | G vs. A |  | 0.249 | 0.85(0.64-1.12) |  | 0.550 | 0.90(0.62-1.29) |
| **rs72855279** |  | 875/873 |  |  | 748/739 |  |  |
| Gender^a^ |  |  |  |  |  |  |  |
| Male | AA | 348/351 |  | 1(Ref) | 352/352 |  | 1(Ref) |
|  | AG | 132/124 | 0.386 | 1.14(0.84-1.55) | 142/125 | 0.382 | 1.14(0.85-1.52) |
|  | GG | 12/15 | 0.639 | 0.82(0.36-1.87) | 11/15 | 0.447 | 0.73(0.32-1.65) |
|  | AG+GG vs. AA |  | 0.494 | 1.11(0.83-1.48) |  | 0.527 | 1.10(0.83-1.45) |
|  | GG vs. AG+AA |  | 0.577 | 0.79(0.35-1.79) |  | 0.395 | 0.70(0.31-1.58) |
|  | G vs. A |  | 0.672 | 1.06(0.82-1.37) |  | 0.769 | 1.04(0.81-1.33) |
| Female | AA | 266/273 |  | 1(Ref) | 176/183 |  | 1(Ref) |
|  | AG | 105/98 | 0.639 | 1.08(0.77-1.52) | 62/57 | 0.747 | 1.07(0.70-1.65) |
|  | GG | 12/12 | 0.712 | 1.17(0.50-2.76) | 5/7 | 0.582 | 0.71(0.21-2.37) |
|  | AG+GG vs. AA |  | 0.587 | 1.09(0.79-1.51) |  | 0.873 | 1.03(0.68-1.57) |
|  | GG vs. AG+AA |  | 0.752 | 1.15(0.49-2.68) |  | 0.570 | 0.71(0.21-2.35) |
|  | G vs. A |  | 0.562 | 1.09(0.82-1.44) |  | 0.972 | 0.99(0.69-1.43) |
| Age^a^ |  |  |  |  |  |  |  |
| ≤60 | AA | 430/451 |  | 1(Ref) | 339/362 |  | 1(Ref) |
|  | AG | 171/166 | 0.519 | 1.09(0.84-1.42) | 128/126 | 0.702 | 1.06(0.79-1.42) |
|  | GG | 16/21 | 0.953 | 0.98(0.49-1.95) | 11/16 | 0.583 | 0.80(0.36-1.78) |
|  | AG+GG vs. AA |  | 0.561 | 1.08(0.84-1.39) |  | 0.830 | 1.03(0.78-1.37) |
|  | GG vs. AG+AA |  | 0.893 | 0.95(0.48-1.89) |  | 0.561 | 0.79(0.36-1.75) |
|  | G vs. A |  | 0.642 | 1.05(0.85-1.31) |  | 0.997 | 1.00(0.78-1.28) |
| >60 | AA | 184/173 |  | 1(Ref) | 189/173 |  | 1(Ref) |
|  | AG | 66/56 | 0.451 | 1.19(0.76-1.85) | 76/56 | 0.340 | 1.23(0.81-1.87) |
|  | GG | 8/6 | 0.767 | 0.84(0.26-2.70) | 5/6 | 0.364 | 0.56(0.16-1.97) |
|  | AG+GG vs. AA |  | 0.535 | 1.14(0.75-1.75) |  | 0.494 | 1.15(0.77-1.74) |
|  | GG vs. AG+AA |  | 0.710 | 0.80(0.25-2.57) |  | 0.303 | 0.51(0.15-1.82) |
|  | G vs. A |  | 0.669 | 1.09(0.75-1.58) |  | 0.755 | 1.06(0.74-1.52) |
| *H.pylori* Infection^a^ |  |  |  |  |  |  |  |
| Positive | AA | 364/174 |  | 1(Ref) | 266/148 |  | 1(Ref) |
|  | AG | 134/62 | 0.761 | 1.06(0.74-1.50) | 109/51 | 0.338 | 1.21(0.82-1.79) |
|  | GG | 10/9 | 0.151 | 0.51(0.20-1.28) | 7/7 | 0.241 | 0.53(0.18-1.54) |
|  | AG+GG vs. AA |  | 0.933 | 0.99(0.70-1.38) |  | 0.542 | 1.12(0.77-1.64) |
|  | GG vs. AG+AA |  | 0.141 | 0.50(0.20-1.26) |  | 0.192 | 0.49(0.17-1.43) |
|  | G vs. A |  | 0.597 | 0.92(0.69-1.24) |  | 0.880 | 1.03(0.74-1.43) |
| Negative | AA | 250/450 |  | 1(Ref) | 262/387 |  | 1(Ref) |
|  | AG | 103/160 | 0.311 | 1.16(0.87-1.56) | 95/131 | 0.681 | 1.07(0.78-1.45) |
|  | GG | 14/18 | 0.326 | 1.43(0.70-2.94) | 9/15 | 0.783 | 0.89(0.38-2.06) |
|  | AG+GG vs. AA |  | 0.224 | 1.19(0.90-1.58) |  | 0.756 | 1.05(0.78-1.41) |
|  | GG vs. AG+AA |  | 0.389 | 1.37(0.67-2.79) |  | 0.752 | 0.87(0.38-2.02) |
|  | G vs. A |  | 0.177 | 1.18(0.93-1.51) |  | 0.863 | 1.02(0.79-1.33) |
| Smoking^b^ |  |  |  |  |  |  |  |
| Ever Smoker | AA | 117/138 |  | 1(Ref) | 113/137 |  | 1(Ref) |
|  | AG | 51/63 | 0.907 | 0.97(0.60-1.56) | 37/61 | 0.164 | 0.69(0.41-1.16) |
|  | GG | 5/4 | 0.738 | 1.27(0.31-5.26) | 2/4 | 0.517 | 0.56(0.09-3.29) |
|  | AG+GG vs. AA |  | 0.971 | 0.99(0.63-1.57) |  | 0.138 | 0.68(0.41-1.13) |
|  | GG vs. AG+AA |  | 0.735 | 1.28(0.31-5.25) |  | 0.546 | 0.58(0.10-3.45) |
|  | G vs. A |  | 0.948 | 1.01(0.68-1.51) |  | 0.141 | 0.71(0.45-1.12) |
| Never Smoker | AA | 274/277 |  | 1(Ref) | 132/225 |  | 1(Ref) |
|  | AG | 92/99 | 0.646 | 0.92(0.64-1.32) | 55/71 | 0.399 | 1.21(0.78-1.87) |
|  | GG | 9/15 | 0.958 | 0.98(0.39-2.42) | 4/11 | 0.371 | 0.57(0.17-1.96) |
|  | AG+GG vs. AA |  | 0.628 | 0.92(0.65-1.30) |  | 0.590 | 1.12(0.74-1.71) |
|  | GG vs. AG+AA |  | 0.898 | 0.94(0.39-2.29) |  | 0.314 | 0.54(0.16-1.81) |
|  | G vs. A |  | 0.641 | 0.93(0.69-1.26) |  | 0.895 | 1.03(0.71-1.48) |
| Drinking^b^ |  |  |  |  |  |  |  |
| Drinker | AA | 84/108 |  | 1(Ref) | 76/108 |  | 1(Ref) |
|  | AG | 36/37 | 0.357 | 1.30(0.74-2.30) | 33/36 | 0.610 | 1.16(0.65-2.09) |
|  | GG | 6/4 | 0.313 | 2.04(0.51-8.11) | 2/4 | 0.554 | 0.58(0.09-3.57) |
|  | AG+GG vs. AA |  | 0.254 | 1.37(0.80-2.34) |  | 0.723 | 1.11(0.63-1.95) |
|  | GG vs. AG+AA |  | 0.363 | 1.86(0.49-7.03) |  | 0.534 | 0.57(0.09-3.40) |
|  | G vs. A |  | 0.191 | 1.36(0.86-2.16) |  | 0.898 | 1.03(0.63-1.70) |
| Nondrinker | AA | 307/308 |  | 1(Ref) | 141/255 |  | 1(Ref) |
|  | AG | 106/123 | 0.271 | 0.83(0.59-1.16) | 50/94 | 0.788 | 0.94(0.62-1.45) |
|  | GG | 8/15 | 0.567 | 0.76(0.30-1.95) | 4/11 | 0.469 | 0.63(0.18-2.20) |
|  | AG+GG vs. AA |  | 0.237 | 0.82(0.60-1.14) |  | 0.669 | 0.91(0.60-1.38) |
|  | GG vs. AG+AA |  | 0.622 | 0.79(0.31-2.01) |  | 0.490 | 0.65(0.19-2.23) |
|  | G vs. A |  | 0.234 | 0.84(0.63-1.12) |  | 0.554 | 0.90(0.62-1.29) |
| **rs7747696** |  | 872/876 |  |  | 746/742 |  |  |
| Gender^a^ |  |  |  |  |  |  |  |
| Male | AA | 255/276 |  | 1(Ref) | 272/277 |  | 1(Ref) |
|  | AG | 205/175 | 0.079 | 1.29(0.97-1.70) | 200/176 | 0.510 | 1.10(0.84-1.44) |
|  | GG | 32/41 | 0.834 | 0.95(0.56-1.60) | 31/41 | 0.272 | 0.75(0.45-1.25) |
|  | AG+GG vs. AA |  | 0.142 | 1.22(0.94-1.59) |  | 0.823 | 1.03(0.80-1.33) |
|  | GG vs. AG+AA |  | 0.511 | 0.84(0.51-1.40) |  | 0.205 | 0.72(0.44-1.19) |
|  | G vs. A |  | 0.371 | 1.10(0.89-1.36) |  | 0.726 | 0.96(0.78-1.18) |
| Female | AA | 201/218 |  | 1(Ref) | 134/145 |  | 1(Ref) |
|  | AG | 145/138 | 0.519 | 1.11(0.81-1.52) | 96/88 | 0.493 | 1.14(0.78-1.68) |
|  | GG | 34/28 | 0.258 | 1.38(0.79-2.42) | 13/15 | 0.630 | 0.82(0.36-1.85) |
|  | AG+GG vs. AA |  | 0.353 | 1.15(0.86-1.55) |  | 0.618 | 1.10(0.76-1.59) |
|  | GG vs. AG+AA |  | 0.329 | 1.31(0.76-2.26) |  | 0.568 | 0.79(0.36-1.76) |
|  | G vs. A |  | 0.243 | 1.15(0.91-1.46) |  | 0.850 | 1.03(0.76-1.39) |
| Age^a^ |  |  |  |  |  |  |  |
| ≤60 | AA | 313/355 |  | 1(Ref) | 254/283 |  | 1(Ref) |
|  | AG | 253/235 | 0.128 | 1.21(0.95-1.54) | 192/186 | 0.479 | 1.10(0.84-1.44) |
|  | GG | 49/51 | 0.358 | 1.23(0.79-1.90) | 30/38 | 0.573 | 0.86(0.51-1.45) |
|  | AG+GG vs. AA |  | 0.102 | 1.21(0.96-1.53) |  | 0.648 | 1.06(0.82-1.37) |
|  | GG vs. AG+AA |  | 0.563 | 1.13(0.74-1.74) |  | 0.460 | 0.83(0.50-1.37) |
|  | G vs. A |  | 0.123 | 1.15(0.96-1.39) |  | 0.950 | 1.01(0.82-1.24) |
| >60 | AA | 143/139 |  | 1(Ref) | 152/139 |  | 1(Ref) |
|  | AG | 97/78 | 0.351 | 1.21(0.81-1.82) | 104/78 | 0.542 | 1.13(0.76-1.67) |
|  | GG | 17/18 | 0.432 | 0.73(0.33-1.60) | 14/18 | 0.216 | 0.61(0.28-1.34) |
|  | AG+GG vs. AA |  | 0.552 | 1.12(0.76-1.65) |  | 0.870 | 1.03(0.71-1.50) |
|  | GG vs. AG+AA |  | 0.353 | 0.70(0.33-1.48) |  | 0.179 | 0.59(0.28-1.27) |
|  | G vs. A |  | 0.926 | 1.02(0.74-1.39) |  | 0.682 | 0.94(0.69-1.27) |
| *H.pylori* Infection^a^ |  |  |  |  |  |  |  |
| Positive | AA | 271/134 |  | 1(Ref) | 196/109 |  | 1(Ref) |
|  | AG | 202/95 | 0.740 | 1.06(0.77-1.46) | 160/83 | 0.681 | 1.08(0.76-1.54) |
|  | GG | 34/18 | 0.876 | 0.95(0.52-1.76) | 24/16 | 0.610 | 0.84(0.43-1.65) |
|  | AG+GG vs. AA |  | 0.808 | 1.04(0.77-1.41) |  | 0.827 | 1.04(0.74-1.46) |
|  | GG vs. AG+AA |  | 0.800 | 0.93(0.51-1.68) |  | 0.531 | 0.81(0.42-1.57) |
|  | G vs. A |  | 0.927 | 1.01(0.79-1.29) |  | 0.938 | 0.99(0.76-1.29) |
| Negative | AA | 185/360 |  | 1(Ref) | 210/313 |  | 1(Ref) |
|  | AG | 148/218 | **0.043(0.301)** | **1.33(1.01-1.75)** | 136/181 | 0.423 | 1.12(0.85-1.49) |
|  | GG | 32/51 | 0.368 | 1.25(0.77-2.01) | 20/40 | 0.287 | 0.74(0.42-1.30) |
|  | AG+GG vs. AA |  | **0.041(0.287)** | **1.31(1.01-1.70)** |  | 0.709 | 1.05(0.80-1.38) |
|  | GG vs. AG+AA |  | 0.670 | 1.11(0.70-1.76) |  | 0.221 | 0.71(0.41-1.23) |
|  | G vs. A |  | 0.069 | 1.21(0.99-1.48) |  | 0.841 | 0.98(0.79-1.22) |
| Smoking^b^ |  |  |  |  |  |  |  |
| Ever Smoker | AA | 86/113 |  | 1(Ref) | 85/112 |  | 1(Ref) |
|  | AG | 71/81 | 0.584 | 1.14(0.72-1.79) | 57/79 | 0.564 | 0.87(0.54-1.40) |
|  | GG | 14/12 | 0.179 | 1.82(0.76-4.35) | 9/12 | 0.784 | 1.14(0.44-3.00) |
|  | AG+GG vs. AA |  | 0.372 | 1.22(0.79-1.88) |  | 0.650 | 0.90(0.57-1.43) |
|  | GG vs. AG+AA |  | 0.212 | 1.72(0.74-4.00) |  | 0.741 | 1.17(0.45-3.04) |
|  | G vs. A |  | 0.226 | 1.24(0.88-1.74) |  | 0.811 | 0.96(0.66-1.39) |
| Never Smoker | AA | 202/218 |  | 1(Ref) | 100/175 |  | 1(Ref) |
|  | AG | 145/143 | 0.776 | 1.05(0.76-1.46) | 80/111 | 0.443 | 1.17(0.79-1.74) |
|  | GG | 28/32 | 0.537 | 1.21(0.66-2.21) | 10/23 | 0.261 | 0.61(0.26-1.44) |
|  | AG+GG vs. AA |  | 0.674 | 1.07(0.79-1.46) |  | 0.695 | 1.08(0.74-1.58) |
|  | GG vs. AG+AA |  | 0.689 | 1.12(0.64-1.99) |  | 0.254 | 0.63(0.28-1.40) |
|  | G vs. A |  | 0.612 | 1.07(0.83-1.36) |  | 0.881 | 0.98(0.72-1.33) |
| Drinking^b^ |  |  |  |  |  |  |  |
| Drinker | AA | 57/84 |  | 1(Ref) | 53/84 |  | 1(Ref) |
|  | AG | 55/56 | 0.122 | 1.51(0.90-2.56) | 47/55 | 0.400 | 1.26(0.73-2.17) |
|  | GG | 13/10 | 0.052 | 2.57(0.99-6.66) | 8/10 | 0.711 | 1.22(0.43-3.47) |
|  | AG+GG vs. AA |  | 0.055 | 1.63(0.99-2.67) |  | 0.374 | 1.27(0.75-2.13) |
|  | GG vs. AG+AA |  | 0.136 | 1.97(0.81-4.81) |  | 0.760 | 1.17(0.43-3.19) |
|  | G vs. A |  | **0.031(0.217)** | **1.53(1.04-2.25)** |  | 0.407 | 1.19(0.79-1.79) |
| Nondrinker | AA | 230/248 |  | 1(Ref) | 110/204 |  | 1(Ref) |
|  | AG | 161/166 | 0.868 | 0.97(0.72-1.33) | 78/133 | 0.953 | 1.01(0.69-1.49) |
|  | GG | 29/34 | 0.746 | 1.10(0.61-1.98) | 8/25 | 0.186 | 0.54(0.22-1.34) |
|  | AG+GG vs. AA |  | 0.953 | 0.99(0.74-1.33) |  | 0.761 | 0.94(0.65-1.37) |
|  | GG vs. AG+AA |  | 0.806 | 1.07(0.61-1.88) |  | 0.199 | 0.57(0.24-1.35) |
|  | G vs. A |  | 0.956 | 1.01(0.80-1.27) |  | 0.457 | 0.89(0.66-1.21) |
| **rs7748341** |  | 872/872 |  |  | 748/738 |  |  |
| Gender^a^ |  |  |  |  |  |  |  |
| Male | AA | 337/337 |  | 1(Ref) | 342/338 |  | 1(Ref) |
|  | AG | 138/135 | 0.581 | 1.09(0.81-1.47) | 145/136 | 0.779 | 1.04(0.78-1.39) |
|  | GG | 16/17 | 0.871 | 0.94(0.45-1.98) | 19/17 | 0.751 | 1.12(0.56-2.23) |
|  | AG+GG vs. AA |  | 0.643 | 1.07(0.80-1.42) |  | 0.724 | 1.05(0.80-1.38) |
|  | GG vs. AG+AA |  | 0.822 | 0.92(0.44-1.92) |  | 0.780 | 1.10(0.56-2.19) |
|  | G vs. A |  | 0.745 | 1.04(0.81-1.34) |  | 0.689 | 1.05(0.83-1.33) |
| Female | AA | 255/265 |  | 1(Ref) | 168/177 |  | 1(Ref) |
|  | AG | 111/104 | 0.576 | 1.10(0.79-1.53) | 68/63 | 0.739 | 1.07(0.71-1.63) |
|  | GG | 15/14 | 0.594 | 1.24(0.57-2.71) | 6/7 | 0.750 | 0.83(0.27-2.61) |
|  | AG+GG vs. AA |  | 0.510 | 1.11(0.81-1.53) |  | 0.816 | 1.05(0.70-1.57) |
|  | GG vs. AG+AA |  | 0.668 | 1.19(0.55-2.57) |  | 0.737 | 0.82(0.26-2.58) |
|  | G vs. A |  | 0.473 | 1.11(0.84-1.45) |  | 0.920 | 1.02(0.72-1.45) |
| Age^a^ |  |  |  |  |  |  |  |
| ≤60 | AA | 411/436 |  | 1(Ref) | 331/349 |  | 1(Ref) |
|  | AG | 184/178 | 0.430 | 1.11(0.86-1.43) | 132/138 | 0.869 | 0.98(0.73-1.30) |
|  | GG | 21/24 | 0.765 | 1.10(0.59-2.06) | 16/17 | 0.933 | 1.03(0.51-2.11) |
|  | AG+GG vs. AA |  | 0.421 | 1.11(0.87-1.42) |  | 0.899 | 0.98(0.74-1.30) |
|  | GG vs. AG+AA |  | 0.859 | 1.06(0.57-1.96) |  | 0.915 | 1.04(0.51-2.11) |
|  | G vs. A |  | 0.451 | 1.09(0.88-1.34) |  | 0.941 | 0.99(0.78-1.26) |
| >60 | AA | 181/166 |  | 1(Ref) | 179/166 |  | 1(Ref) |
|  | AG | 65/61 | 0.873 | 1.04(0.67-1.61) | 81/61 | 0.372 | 1.21(0.80-1.83) |
|  | GG | 10/7 | 0.836 | 0.89(0.31-2.60) | 9/7 | 0.982 | 1.01(0.36-2.89) |
|  | AG+GG vs. AA |  | 0.939 | 1.02(0.67-1.55) |  | 0.404 | 1.18(0.80-1.76) |
|  | GG vs. AG+AA |  | 0.793 | 0.87(0.30-2.52) |  | 0.916 | 0.95(0.33-2.71) |
|  | G vs. A |  | 0.982 | 1.00(0.69-1.44) |  | 0.490 | 1.13(0.80-1.59) |
| *H.pylori* Infection^a^ |  |  |  |  |  |  |  |
| Positive | AA | 351/169 |  | 1(Ref) | 254/143 |  | 1(Ref) |
|  | AG | 141/67 | 0.832 | 1.04(0.74-1.47) | 117/56 | 0.364 | 1.19(0.82-1.75) |
|  | GG | 15/10 | 0.421 | 0.71(0.31-1.63) | 11/8 | 0.559 | 0.76(0.30-1.93) |
|  | AG+GG vs. AA |  | 0.971 | 0.99(0.71-1.38) |  | 0.496 | 1.14(0.79-1.64) |
|  | GG vs. AG+AA |  | 0.400 | 0.70(0.31-1.60) |  | 0.460 | 0.70(0.28-1.79) |
|  | G vs. A |  | 0.754 | 0.96(0.72-1.27) |  | 0.728 | 1.06(0.77-1.45) |
| Negative | AA | 241/433 |  | 1(Ref) | 256/372 |  | 1(Ref) |
|  | AG | 108/172 | 0.389 | 1.14(0.85-1.51) | 96/143 | 0.855 | 0.97(0.72-1.32) |
|  | GG | 16/21 | 0.314 | 1.41(0.72-2.76) | 14/16 | 0.534 | 1.26(0.61-2.64) |
|  | AG+GG vs. AA |  | 0.285 | 1.16(0.88-1.53) |  | 0.996 | 1.00(0.75-1.34) |
|  | GG vs. AG+AA |  | 0.391 | 1.34(0.69-2.60) |  | 0.513 | 1.28(0.61-2.65) |
|  | G vs. A |  | 0.222 | 1.16(0.92-1.47) |  | 0.819 | 1.03(0.80-1.33) |
| Smoking^b^ |  |  |  |  |  |  |  |
| Ever Smoker | AA | 113/135 |  | 1(Ref) | 110/134 |  | 1(Ref) |
|  | AG | 53/67 | 0.861 | 0.96(0.60-1.53) | 37/65 | 0.118 | 0.66(0.39-1.11) |
|  | GG | 7/4 | 0.243 | 2.19(0.59-8.16) | 6/4 | 0.427 | 1.72(0.45-6.50) |
|  | AG+GG vs. AA |  | 0.903 | 1.03(0.65-1.62) |  | 0.211 | 0.73(0.45-1.20) |
|  | GG vs. AG+AA |  | 0.235 | 2.21(0.60-8.21) |  | 0.358 | 1.87(0.49-7.13) |
|  | G vs. A |  | 0.637 | 1.10(0.75-1.62) |  | 0.432 | 0.84(0.55-1.29) |
| Never Smoker | AA | 263/269 |  | 1(Ref) | 125/218 |  | 1(Ref) |
|  | AG | 101/108 | 0.741 | 0.94(0.66-1.34) | 59/79 | 0.515 | 1.15(0.75-1.76) |
|  | GG | 11/16 | 0.893 | 1.06(0.45-2.51) | 6/12 | 0.735 | 0.83(0.28-2.43) |
|  | AG+GG vs. AA |  | 0.744 | 0.95(0.68-1.32) |  | 0.632 | 1.11(0.73-1.66) |
|  | GG vs. AG+AA |  | 0.983 | 1.01(0.44-2.32) |  | 0.623 | 0.77(0.27-2.20) |
|  | G vs. A |  | 0.783 | 0.96(0.72-1.28) |  | 0.810 | 1.04(0.73-1.49) |
| Drinking^b^ |  |  |  |  |  |  |  |
| Drinker | AA | 78/104 |  | 1(Ref) | 74/104 |  | 1(Ref) |
|  | AG | 40/42 | 0.310 | 1.33(0.77-2.30) | 31/41 | 0.991 | 1.00(0.56-1.78) |
|  | GG | 7/4 | 0.145 | 2.71(0.71-10.37) | 6/4 | 0.370 | 1.87(0.48-7.39) |
|  | AG+GG vs. AA |  | 0.182 | 1.43(0.85-2.40) |  | 0.785 | 1.08(0.62-1.87) |
|  | GG vs. AG+AA |  | 0.194 | 2.36(0.65-8.60) |  | 0.356 | 1.88(0.49-7.22) |
|  | G vs. A |  | 0.109 | 1.44(0.92-2.24) |  | 0.570 | 1.15(0.72-1.84) |
| Nondrinker | AA | 297/301 |  | 1(Ref) | 135/249 |  | 1(Ref) |
|  | AG | 114/131 | 0.345 | 0.85(0.62-1.19) | 55/101 | 0.695 | 0.92(0.61-1.40) |
|  | GG | 11/16 | 0.944 | 0.97(0.41-2.28) | 5/12 | 0.703 | 0.80(0.26-2.50) |
|  | AG+GG vs. AA |  | 0.357 | 0.86(0.63-1.18) |  | 0.638 | 0.91(0.61-1.36) |
|  | GG vs. AG+AA |  | 0.971 | 0.98(0.43-2.28) |  | 0.707 | 0.81(0.26-2.49) |
|  | G vs. A |  | 0.418 | 0.89(0.68-1.17) |  | 0.597 | 0.91(0.64-1.29) |
| **rs7749023** |  | 871/872 |  |  | 747/740 |  |  |
| Gender^a^ |  |  |  |  |  |  |  |
| Male | AA | 286/288 |  | 1(Ref) | 288/289 |  | 1(Ref) |
|  | AC | 177/168 | 0.512 | 1.10(0.83-1.46) | 192/169 | 0.607 | 1.07(0.82-1.41) |
|  | CC | 28/34 | 0.719 | 0.90(0.51-1.58) | 25/34 | 0.277 | 0.74(0.42-1.28) |
|  | AC+CC vs. AA |  | 0.648 | 1.07(0.81-1.39) |  | 0.888 | 1.02(0.79-1.32) |
|  | CC vs. AC+AA |  | 0.605 | 0.87(0.50-1.50) |  | 0.230 | 0.71(0.41-1.24) |
|  | C vs. A |  | 0.869 | 1.02(0.82-1.27) |  | 0.722 | 0.96(0.80-1.19) |
| Female | AA | 215/235 |  | 1(Ref) | 140/158 |  | 1(Ref) |
|  | AC | 140/124 | 0.241 | 1.21(0.88-1.66) | 89/77 | 0.245 | 1.26(0.85-1.87) |
|  | CC | 25/23 | 0.457 | 1.27(0.68-2.36) | 13/13 | 0.988 | 1.01(0.44-2.32) |
|  | AC+CC vs. AA |  | 0.205 | 1.22(0.90-1.64) |  | 0.285 | 1.23(0.84-1.79) |
|  | CC vs. AC+AA |  | 0.614 | 1.17(0.64-2.15) |  | 0.877 | 0.94(0.41-2.12) |
|  | C vs. A |  | 0.215 | 1.17(0.91-1.50) |  | 0.408 | 1.14(0.84-1.56) |
| Age^a^ |  |  |  |  |  |  |  |
| ≤60 | AA | 346/378 |  | 1(Ref) | 273/302 |  | 1(Ref) |
|  | AC | 233/217 | 0.194 | 0.18(0.92-1.51) | 180/171 | 0.407 | 1.12(0.85-1.47) |
|  | CC | 36/42 | 0.686 | 1.11(0.68-1.80) | 25/32 | 0.620 | 0.87(0.50-1.52) |
|  | AC+CC vs. AA |  | 0.199 | 1.17(0.92-1.47) |  | 0.543 | 1.08(0.84-1.41) |
|  | CC vs. AC+AA |  | 0.876 | 1.04(0.65-1.67) |  | 0.509 | 0.83(0.48-1.44) |
|  | C vs. A |  | 0.267 | 1.11(0.92-1.35) |  | 0.810 | 1.03(0.83-1.27) |
| >60 | AA | 155/145 |  | 1(Ref) | 155/145 |  | 1(Ref) |
|  | AC | 84/75 | 0.782 | 1.06(0.70-1.60) | 101/75 | 0.527 | 1.14(0.77-1.69) |
|  | CC | 17/15 | 0.477 | 0.74(0.33-1.68) | 13/15 | 0.371 | 0.69(0.30-1.56) |
|  | AC+CC vs. AA |  | 0.962 | 1.01(0.68-1.49) |  | 0.756 | 1.06(0.73-1.55) |
|  | CC vs. AC+AA |  | 0.500 | 0.76(0.35-1.67) |  | 0.317 | 0.66(0.30-1.48) |
|  | C vs. A |  | 0.812 | 0.96(0.70-1.33) |  | 0.896 | 0.98(0.72-1.34) |
| *H.pylori* Infection^a^ |  |  |  |  |  |  |  |
| Positive | AA | 297/144 |  | 1(Ref) | 206/119 |  | 1(Ref) |
|  | AC | 181/86 | 0.843 | 1.03(0.75-1.43) | 156/74 | 0.272 | 1.22(0.85-1.75) |
|  | CC | 27/15 | 0.631 | 0.85(0.44-1.66) | 19/14 | 0.488 | 0.77(0.37-1.60) |
|  | AC+CC vs. AA |  | 0.972 | 1.01(0.74-1.37) |  | 0.421 | 1.15(0.82-1.62) |
|  | CC vs. AC+AA |  | 0.604 | 0.84(0.44-1.62) |  | 0.351 | 0.71(0.35-1.46) |
|  | C vs. A |  | 0.866 | 0.98(0.76-1.26) |  | 0.763 | 1.04(0.79-1.38) |
| Negative | AA | 204/379 |  | 1(Ref) | 222/328 |  | 1(Ref) |
|  | AC | 136/206 | 0.132 | 1.24(0.94-1.63) | 125/172 | 0.633 | 1.07(0.81-1.43) |
|  | CC | 26/42 | 0.515 | 1.19(0.71-2.00) | 19/33 | 0.560 | 0.84(0.46-1.52) |
|  | AC+CC vs. AA |  | 0.128 | 1.23(0.94-1.59) |  | 0.800 | 1.04(0.79-1.36) |
|  | CC vs. AC+AA |  | 0.757 | 1.08(0.65-1.80) |  | 0.516 | 0.83(0.46-1.48) |
|  | C vs. A |  | 0.171 | 1.16(0.94-1.43) |  | 0.962 | 0.99(0.79-1.25) |
| Smoking^b^ |  |  |  |  |  |  |  |
| Ever Smoker | AA | 94/116 |  | 1(Ref) | 92/115 |  | 1(Ref) |
|  | AC | 66/82 | 0.939 | 0.98(0.63-1.54) | 54/80 | 0.269 | 0.76(0.47-1.23) |
|  | CC | 13/8 | 0.088 | 2.33(0.88-6.14) | 7/8 | 0.760 | 1.19(0.39-3.58) |
|  | AC+CC vs. AA |  | 0.678 | 1.10(0.71-1.69) |  | 0.340 | 0.80(0.50-1.27) |
|  | CC vs. AC+AA |  | 0.083 | 2.33(0.90-6.03) |  | 0.673 | 1.27(0.42-3.81) |
|  | C vs. A |  | 0.320 | 1.19(0.84-1.69) |  | 0.519 | 0.88(0.60-1.29) |
| Never Smoker | AA | 223/236 |  | 1(Ref) | 108/190 |  | 1(Ref) |
|  | AC | 132/128 | 0.749 | 1.06(0.76-1.47) | 73/99 | 0.418 | 1.18(0.79-1.77) |
|  | CC | 19/29 | 0.819 | 0.93(0.47-1.81) | 9/20 | 0.365 | 0.66(0.27-1.61) |
|  | AC+CC vs. AA |  | 0.871 | 1.03(0.75-1.41) |  | 0.622 | 1.10(0.75-1.62) |
|  | CC vs. AC+AA |  | 0.651 | 0.86(0.45-1.64) |  | 0.353 | 0.67(0.29-1.56) |
|  | C vs. A |  | 0.960 | 0.99(0.77-1.29) |  | 0.971 | 1.01(0.73-1.38) |
| Drinking^b^ |  |  |  |  |  |  |  |
| Drinker | AA | 63/91 |  | 1(Ref) | 60/91 |  | 1(Ref) |
|  | AC | 51/51 | 0.148 | 1.47(0.87-2.49) | 44/50 | 0.437 | 1.24(0.72-2.14) |
|  | CC | 12/8 | 0.051 | 2.74(1.00-7.55) | 7/8 | 0.690 | 1.26(0.40-3.93) |
|  | AC+CC vs. AA |  | 0.061 | 1.61(0.98-2.64) |  | 0.403 | 1.25(0.74-2.10) |
|  | CC vs. AC+AA |  | 0.110 | 2.19(0.84-5.71) |  | 0.742 | 1.20(0.40-3.58) |
|  | C vs. A |  | **0.029(0.203)** | **1.55(1.05-2.31)** |  | 0.419 | 1.19(0.78-1.82) |
| Nondrinker | AA | 253/262 |  | 1(Ref) | 116/215 |  | 1(Ref) |
|  | AC | 147/157 | 0.604 | 0.92(0.67-1.26) | 72/127 | 0.881 | 0.97(0.66-1.44) |
|  | CC | 20/29 | 0.743 | 0.90(0.46-1.73) | 7/20 | 0.282 | 0.59(0.23-1.54) |
|  | AC+CC vs. AA |  | 0.561 | 0.92(0.68-1.23) |  | 0.684 | 0.92(0.63-1.35) |
|  | CC vs. AC+AA |  | 0.739 | 0.90(0.47-1.70) |  | 0.325 | 0.63(0.25-1.59) |
|  | C vs. A |  | 0.544 | 0.93(0.73-1.18) |  | 0.488 | 0.89(0.65-1.23) |
| Note: ^a^, *P* was adjusted by the other two factors of gender, age and *H.pylori* infection status; ^b^, *P* was adjusted by gender, age and *H.pylori* infection status; AG, atrophic gastritis; GC, gastric cancer; CON, control; OR, odds ratio; CI, confidence interval; *P*_corr_, *P* values after Bonferroni correction. The results are in bold if *P*<0.05. | | | | | | | |
|  |  |  |  |  |  |  |  |
|  |  |  |  |  |  |  |  |
