# Supplementary material for: Long non-coding RNA polymorphisms in 6p21.1 are associated with atrophic gastritis risk and gastric cancer prognosis
Source: Oncotarget. 2017 Aug 10;8(56):95303–15. doi: 10.18632/oncotarget.20115 (PMC5707023; doi:10.18632/oncotarget.20115)
Supplement: Supplementary file 4 [file oncotarget-08-95303-s004.docx]

| Table S5. The interaction effects between the lncRNA SNPs and environmental factors on GC risk | | | | | | |  |
| --- | --- | --- | --- | --- | --- | --- | --- |
| SNP genotypes | *H.pylori* Infection^a^ | | Smoking^b^ | | Drinking^b^ | | |
|  | Negative | Positive | No | Yes | No | Yes | |
| **rs61516247** | n=901 | n=590 | n=500 | n=356 | n=558 | n=260 | |
| GA+GG |  |  |  |  |  |  | |
| Case/Control | 335/495 | 351/194 | 176/290 | 142/188 | 180/338 | 105/139 | |
| OR(95%CI) | 1(Ref) | 2.67(2.14-3.34) | 1(Ref) | 1.25(0.93-1.66) | 1(Ref) | 1.42(1.04-1.94) | |
| AA |  |  |  |  |  |  | |
| Case/Control | 31/40 | 32/13 | 15/19 | 11/15 | 16/24 | 6/10 | |
| OR(95%CI) | 1.15(0.70-1.87) | 3.64(1.89-7.03) | 1.30(0.64-2.63) | 1.21(0.54-2.69) | 1.25(0.65-2.42) | 1.42(1.04-1.94) | |
|  | *P*_interaction_=0.729 | | *P*_interaction_=0.361 | | *P*_interaction_=0.250 | | |
| **rs1886753** | n=897 | n=590 | n=499 | n=354 | n=556 | n=259 | |
| GG |  |  |  |  |  |  | |
| Case/Control | 73/120 | 88/47 | 44/75 | 28/47 | 44/85 | 24/37 | |
| OR(95%CI) | 1(Ref) | 3.08(1.95-4.87) | 1(Ref) | 1.02(0.56-1.85) | 1(Ref) | 1.25(0.67-2.35) | |
| AG+AA |  |  |  |  |  |  | |
| Case/Control | 292/412 | 294/161 | 147/233 | 124/155 | 152/275 | 86/112 | |
| OR(95%CI) | 1.17(0.84-1.62) | 3.00(2.12-4.25) | 1.08(0.70-1.65) | 1.36(0.88-2.12) | 1.07(0.71-1.62) | 1.48(0.94-2.35) | |
|  | *P*_interaction_=0.553 | | *P*_interaction_=0.301 | | *P*_interaction_=0.806 | | |
| **rs80112640** | n=898 | n=587 | n=497 | n=354 | n=555 | n=258 | |
| GG |  |  |  |  |  |  | |
| Case/Control | 9/15 | 7/7 | 4/11 | 2/4 | 4/11 | 2/4 | |
| OR(95%CI) | 1(Ref) | 1.67(0.44-6.33) | 1(Ref) | 1.38(0.18-10.65) | 1(Ref) | 1.38(0.18-10.65) | |
| AG+AA |  |  |  |  |  |  | |
| Case/Control | 356/518 | 373/200 | 185/297 | 149/199 | 190/350 | 107/145 | |
| OR(95%CI) | 1.15(0.50-2.65) | 3.11(1.34-7.23) | 1.71(0.54-5.46) | 2.06(0.64-6.59) | 1.49(0.47-4.75) | 2.03(0.63-6.55) | |
|  | *P*_interaction_=0.430 | | *P*_interaction_=0.981 | | *P*_interaction_=0.844 | | |
| **rs72855279** | n=899 | n=588 | n=498 | n=354 | n=555 | n=259 | |
| GG |  |  |  |  |  |  | |
| Case/Control | 9/15 | 7/7 | 4/11 | 2/4 | 4/11 | 2/4 | |
| OR(95%CI) | 1(Ref) | 1.67(0.44-6.33) | 1(Ref) | 1.38(0.18-10.65) | 1(Ref) | 1.38(0.18-10.65) | |
| AG+AA |  |  |  |  |  |  | |
| Case/Control | 357/518 | 375/199 | 187/296 | 150/198 | 191/349 | 109/144 | |
| OR(95%CI) | 1.15(0.50-2.65) | 3.14(1.35-7.31) | 1.74(0.55-5.54) | 2.08(0.65-6.67) | 1.51(0.47-4.79) | 2.08(0.65-6.71) | |
|  | *P*_interaction_=0.429 | | *P*_interaction_=0.988 | | *P*_interaction_=0.827 | | |
| **rs7747696** | n=900 | n=588 | n=499 | n=354 | n=558 | n=257 | |
| GG |  |  |  |  |  |  | |
| Case/Control | 20/40 | 24/16 | 10/23 | 9/12 | 8/25 | 8/10 | |
| OR(95%CI) | 1(Ref) | 3.00(1.31-6.89) | 1(Ref) | 1.73(0.55-5.39) | 1(Ref) | 2.50(0.74-8.50) | |
| AG+AA |  |  |  |  |  |  | |
| Case/Control | 346/494 | 356/192 | 180/286 | 142/191 | 188/337 | 100/139 | |
| OR(95%CI) | 1.40(0.81-2.44) | 3.71(2.11-6.52) | 1.45(0.67-3.11) | 1.71(0.79-3.71) | 1.74(0.77-3.94) | 2.25(0.97-5.19) | |
|  | *P*_interaction_=0.770 | | *P*_interaction_=0.387 | | *P*_interaction_=0.304 | | |
| **rs7748341** | n=897 | n=589 | n=499 | n=356 | n=557 | n=260 | |
| AA |  |  |  |  |  |  | |
| Case/Control | 256/372 | 254/143 | 125/218 | 110/134 | 135/249 | 74/104 | |
| OR(95%CI) | 1(Ref) | 2.58(1.99-3.35) | 1(Ref) | 1.43(1.03-2.00) | 1(Ref) | 1.31(0.91-1.89) | |
| AG+GG |  |  |  |  |  |  | |
| Case/Control | 110/159 | 128/64 | 65/91 | 43/69 | 60/113 | 37/45 | |
| OR(95%CI) | 1.01(0.75-1.34) | 2.91(2.07-4.08) | 1.25(0.85-1.83) | 1.09(0.70-1.69) | 0.98(0.67-1.43) | 1.52(0.94-2.46) | |
|  | *P*_interaction_=0.615 | | *P*_interaction_=0.196 | | *P*_interaction_=0.668 | | |
| **rs7749023** | n=899 | n=588 | n=499 | n=356 | n=557 | n=260 | |
| CC |  |  |  |  |  |  | |
| Case/Control | 19/33 | 19/14 | 9/20 | 7/8 | 7/20 | 7/8 | |
| OR(95%CI) | 1(Ref) | 2.36(0.97-5.75) | 1(Ref) | 1.94(0.54-7.02) | 1(Ref) | 2.50(0.66-9.46) | |
| AC+AA |  |  |  |  |  |  | |
| Case/Control | 347/500 | 362/193 | 181/289 | 146/195 | 188/342 | 104/141 | |
| OR(95%CI) | 1.21(0.67-2.16) | 3.26(1.80-5.88) | 1.39(0.62-3.12) | 1.66(0.74-3.76) | 1.57(0.65-3.78) | 2.11(0.86-5.17) | |
|  | *P*_interaction_=0.762 | | *P*_interaction_=0.436 | | *P*_interaction_=0.413 | | |
| Note: ^a^, *P* for interaction was adjusted by gender and age; ^b^, *P* for interaction was adjusted by gender, age and *H.pylori* infection status; AG, atrophic gastritis; GC, gastric cancer; CON, control; OR, odds ratio; CI, confidence interval. | | | | | | |  |
|  |  |  |  |  |  |  |  |
