# Supplementary material for: Long non-coding RNA polymorphisms in 6p21.1 are associated with atrophic gastritis risk and gastric cancer prognosis
Source: Oncotarget. 2017 Aug 10;8(56):95303–15. doi: 10.18632/oncotarget.20115 (PMC5707023; doi:10.18632/oncotarget.20115)
Supplement: Supplementary file 5 [file oncotarget-08-95303-s005.docx]

| Table S7. The association between the lncRNA SNPs and clinicopathological parameters^a^ | | | | | | | | | |  |
| --- | --- | --- | --- | --- | --- | --- | --- | --- | --- | --- |
| Parameters | N | SNP genotypes | | | *P*(*P*_corr_) | | | | | |
|  |  | Homozygote wild(%) | Heterozygote(%) | Homozygote variant(%) | Heterozygote vs. homozygote wild | Homozygote variant vs. homozygote wild | Dominant model | Recessive model | Allelic model | |
|  |  |  |  |  |  |  |  |  |  |  |
| **rs61516247** | 353 |  |  |  |  |  |  |  |  | |
| Macroscopic type |  |  |  |  | 0.732 | **0.009(0.063)**^b^ | 0.587 | **0.005(0.035)**^c^ | 0.096 | |
| Borrmann Ⅰ-Ⅱ | 74 | 35(47.3) | 27(36.5) | 12(16.2) |  |  |  |  |  | |
| Borrmann Ⅲ-Ⅳ | 243 | 124(51.0) | 105(43.2) | 14(5.8) |  |  |  |  |  | |
| Lauren classification |  |  |  |  | 0.961 | 0.289 | 0.719 | 0.296 | 0.461 | |
| Intestinal-type | 132 | 68(51.5) | 55(41.7) | 9(6.8) |  |  |  |  |  | |
| Diffuse-type | 217 | 105(48.4) | 91(41.9) | 21(9.7) |  |  |  |  |  | |
| TNM stage |  |  |  |  | 0.102 | **0.045(0.315)**^d^ | **0.036(0.567)**^e^ | 0.115 | **0.021(0.147)**^f^ | |
| Ⅰ-Ⅱ | 176 | 77(43.8) | 80(45.5) | 19(10.8) |  |  |  |  |  | |
| Ⅲ-Ⅳ | 177 | 98(55.4) | 68(38.4) | 11(6.2) |  |  |  |  |  | |
| Lymphatic metastasis |  |  |  |  | 0.658 | 0.297 | 0.462 | 0.367 | 0.337 | |
| Positive | 214 | 110(51.4) | 88(41.1) | 16(7.5) |  |  |  |  |  | |
| Negative | 139 | 65(46.8) | 60(43.2) | 14(10.1) |  |  |  |  |  | |
| Depth of invasion |  |  |  |  | 0.234 | 0.079 | 0.109 | 0.136 | 0.059 | |
| T1+T2 | 83 | 35(42.2) | 38(45.8) | 10(12.0) |  |  |  |  |  | |
| T3+T4 | 182 | 96(52.7) | 74(40.7) | 12(6.6) |  |  |  |  |  | |
| **rs1886753** | 353 |  |  |  |  |  |  |  |  | |
| Macroscopic type |  |  |  |  | 0.091 | **0.014(0.098)**^g^ | **0.030(0.210)**^h^ | 0.107 | **0.020(0.140)**^i^ | |
| Borrmann Ⅰ-Ⅱ | 74 | 26(35.1) | 35(47.3) | 13(17.6) |  |  |  |  |  | |
| Borrmann Ⅲ-Ⅳ | 243 | 56(23.0) | 123(50.6) | 64(26.3) |  |  |  |  |  | |
| Lauren classification |  |  |  |  | 0.835 | 0.974 | 0.899 | 0.937 | 0.975 | |
| Intestinal-type | 132 | 34(25.8) | 68(51.5) | 30(22.7) |  |  |  |  |  | |
| Diffuse-type | 217 | 56(25.8) | 109(50.2) | 52(24.0) |  |  |  |  |  | |
| TNM stage |  |  |  |  | 0.624 | 0.159 | 0.810 | **0.036(0.252)**^j^ | 0.160 | |
| Ⅰ-Ⅱ | 176 | 46(26.1) | 97(55.1) | 33(18.8) |  |  |  |  |  | |
| Ⅲ-Ⅳ | 177 | 45(25.4) | 83(46.9) | 49(27.7) |  |  |  |  |  | |
| Lymphatic metastasis |  |  |  |  | 0.538 | 0.420 | 0.919 | 0.133 | 0.402 | |
| Positive | 214 | 56(26.2) | 103(48.1) | 55(25.7) |  |  |  |  |  | |
| Negative | 139 | 35(25.2) | 77(55.4) | 27(19.4) |  |  |  |  |  | |
| Depth of invasion |  |  |  |  | 0.979 | **0.029(0.203)**^k^ | 0.394 | **0.019(0.133)**^l^ | **0.048(0.336)**^m^ | |
| T1+T2 | 83 | 23(27.7) | 47(56.6) | 13(15.7) |  |  |  |  |  | |
| T3+T4 | 182 | 41(22.5) | 87(47.8) | 54(29.7) |  |  |  |  |  | |
| **rs80112640** | 353 |  |  |  |  |  |  |  |  | |
| Macroscopic type |  |  |  |  | 0.418 | 0.575 | 0.510 | 0.569 | 0.677 | |
| Borrmann Ⅰ-Ⅱ | 74 | 49(66.2) | 24(32.4) | 1(1.4) |  |  |  |  |  | |
| Borrmann Ⅲ-Ⅳ | 243 | 170(70.0) | 67(27.6) | 6(2.5) |  |  |  |  |  | |
| Lauren classification |  |  |  |  | 0.401 | 0.614 | 0.496 | 0.582 | 0.672 | |
| Intestinal-type | 132 | 87(65.9) | 42(31.8) | 3(2.3) |  |  |  |  |  | |
| Diffuse-type | 217 | 149(68.7) | 62(28.6) | 6(2.8) |  |  |  |  |  | |
| TNM stage |  |  |  |  | 0.381 | 0.309 | 0.565 | 0.268 | 0.868 | |
| Ⅰ-Ⅱ | 176 | 122(69.3) | 48(27.3) | 6(3.4) |  |  |  |  |  | |
| Ⅲ-Ⅳ | 177 | 118(66.7) | 56(31.6) | 3(1.7) |  |  |  |  |  | |
| Lymphatic metastasis |  |  |  |  | 0.543 | 0.770 | 0.513 | 0.807 | 0.521 | |
| Positive | 214 | 143(66.8) | 65(30.4) | 6(2.8) |  |  |  |  |  | |
| Negative | 139 | 97(69.8) | 39(28.1) | 3(2.2) |  |  |  |  |  | |
| Depth of invasion |  |  |  |  | 0.449 | 0.577 | 0.570 | 0.513 | 0.768 | |
| T1+T2 | 83 | 60(72.3) | 20(24.1) | 3(3.6) |  |  |  |  |  | |
| T3+T4 | 182 | 126(69.2) | 52(28.6) | 4(2.2) |  |  |  |  |  | |
| **rs72855279** | 352 |  |  |  |  |  |  |  |  | |
| Macroscopic type |  |  |  |  | 0.395 | 0.575 | 0.486 | 0.568 | 0.653 | |
| Borrmann Ⅰ-Ⅱ | 74 | 49(66.2) | 24(32.4) | 1(1.4) |  |  |  |  |  | |
| Borrmann Ⅲ-Ⅳ | 242 | 170(70.2) | 66(27.3) | 6(2.5) |  |  |  |  |  | |
| Lauren classification |  |  |  |  | 0.398 | 0.624 | 0.493 | 0.588 | 0.667 | |
| Intestinal-type | 131 | 87(66.4) | 41(31.3) | 3(2.3) |  |  |  |  |  | |
| Diffuse-type | 217 | 150(69.1) | 61(28.1) | 6(2.8) |  |  |  |  |  | |
| TNM stage |  |  |  |  | 0.489 | 0.303 | 0.695 | 0.271 | 0.994 | |
| Ⅰ-Ⅱ | 176 | 122(69.3) | 48(27.3) | 6(3.4) |  |  |  |  |  | |
| Ⅲ-Ⅳ | 176 | 119(67.6) | 54(30.7) | 3(1.7) |  |  |  |  |  | |
| Lymphatic metastasis |  |  |  |  | 0.649 | 0.779 | 0.612 | 0.803 | 0.606 | |
| Positive | 213 | 144(67.6) | 63(29.6) | 6(2.8) |  |  |  |  |  | |
| Negative | 139 | 97(69.8) | 39(28.1) | 3(2.2) |  |  |  |  |  | |
| Depth of invasion |  |  |  |  | 0.491 | 0.577 | 0.615 | 0.518 | 0.811 | |
| T1+T2 | 83 | 60(72.3) | 20(24.1) | 3(3.6) |  |  |  |  |  | |
| T3+T4 | 181 | 126(69.6) | 51(28.2) | 4(2.2) |  |  |  |  |  | |
| **rs7747696** | 351 |  |  |  |  |  |  |  |  | |
| Macroscopic type |  |  |  |  | 0.680 | 0.928 | 0.715 | 0.884 | 0.830 | |
| Borrmann Ⅰ-Ⅱ | 74 | 37(50.0) | 33(44.6) | 4(5.4) |  |  |  |  |  | |
| Borrmann Ⅲ-Ⅳ | 242 | 125(51.7) | 102(42.1) | 15(6.2) |  |  |  |  |  | |
| Lauren classification |  |  |  |  | 0.274 | 0.144 | 0.560 | 0.088 | 0.821 | |
| Intestinal-type | 130 | 64(49.2) | 61(46.9) | 5(3.8) |  |  |  |  |  | |
| Diffuse-type | 217 | 111(51.2) | 89(41.0) | 17(7.8) |  |  |  |  |  | |
| TNM stage |  |  |  |  | 0.327 | 0.919 | 0.405 | 0.884 | 0.550 | |
| Ⅰ-Ⅱ | 174 | 92(52.9) | 71(40.8) | 11(6.3) |  |  |  |  |  | |
| Ⅲ-Ⅳ | 177 | 86(48.6) | 80(45.2) | 11(6.2) |  |  |  |  |  | |
| Lymphatic metastasis |  |  |  |  | 0.555 | 0.261 | 0.410 | 0.295 | 0.294 | |
| Positive | 214 | 105(49.1) | 93(43.5) | 16(7.5) |  |  |  |  |  | |
| Negative | 137 | 73(53.3) | 58(42.3) | 6(4.4) |  |  |  |  |  | |
| Depth of invasion |  |  |  |  | 0.089 | 0.500 | 0.095 | 0.827 | 0.161 | |
| T1+T2 | 82 | 48(58.5) | 29(35.4) | 5(6.1) |  |  |  |  |  | |
| T3+T4 | 181 | 87(48.1) | 82(45.3) | 12(6.6) |  |  |  |  |  | |
| **rs7748341** | 353 |  |  |  |  |  |  |  |  | |
| Macroscopic type |  |  |  |  | 0.685 | 0.337 | 0.886 | 0.341 | 0.849 | |
| Borrmann Ⅰ-Ⅱ | 74 | 49(66.2) | 24(32.4) | 1(1.4) |  |  |  |  |  | |
| Borrmann Ⅲ-Ⅳ | 243 | 162(66.7) | 72(29.6) | 9(3.7) |  |  |  |  |  | |
| Lauren classification |  |  |  |  | 0.609 | 0.363 | 0.817 | 0.364 | 0.902 | |
| Intestinal-type | 132 | 85(64.4) | 43(32.6) | 4(3.0) |  |  |  |  |  | |
| Diffuse-type | 217 | 141(65.0) | 67(30.9) | 9(4.1) |  |  |  |  |  | |
| TNM stage |  |  |  |  | 0.374 | 0.370 | 0.579 | 0.314 | 0.897 | |
| Ⅰ-Ⅱ | 176 | 117(66.5) | 51(29.0) | 8(4.5) |  |  |  |  |  | |
| Ⅲ-Ⅳ | 177 | 113(63.8) | 59(33.3) | 5(2.8) |  |  |  |  |  | |
| Lymphatic metastasis |  |  |  |  | 0.612 | 0.621 | 0.542 | 0.640 | 0.498 | |
| Positive | 214 | 137(64.0) | 68(31.8) | 9(4.2) |  |  |  |  |  | |
| Negative | 139 | 93(66.9) | 42(30.2) | 4(2.9) |  |  |  |  |  | |
| Depth of invasion |  |  |  |  | 0.350 | 0.822 | 0.427 | 0.723 | 0.583 | |
| T1+T2 | 83 | 58(69.9) | 21(25.3) | 4(4.8) |  |  |  |  |  | |
| T3+T4 | 182 | 119(65.4) | 56(30.8) | 7(3.8) |  |  |  |  |  | |
| **rs7749023** | 353 |  |  |  |  |  |  |  |  | |
| Macroscopic type |  |  |  |  | 0.493 | 0.826 | 0.482 | 0.942 | 0.551 | |
| Borrmann Ⅰ-Ⅱ | 74 | 39(52.7) | 31(41.9) | 4(5.4) |  |  |  |  |  | |
| Borrmann Ⅲ-Ⅳ | 243 | 137(56.4) | 93(38.3) | 13(5.3) |  |  |  |  |  | |
| Lauren classification |  |  |  |  | 0.405 | 0.081 | 0.780 | 0.061 | 0.604 | |
| Intestinal-type | 132 | 71(53.8) | 57(43.2) | 4(3.0) |  |  |  |  |  | |
| Diffuse-type | 217 | 118(54.4) | 83(38.2) | 16(7.4) |  |  |  |  |  | |
| TNM stage |  |  |  |  | 0.236 | 0.541 | 0.245 | 0.712 | 0.284 | |
| Ⅰ-Ⅱ | 176 | 101(57.4) | 66(37.5) | 9(5.1) |  |  |  |  |  | |
| Ⅲ-Ⅳ | 177 | 91(51.4) | 75(42.4) | 11(6.2) |  |  |  |  |  | |
| Lymphatic metastasis |  |  |  |  | 0.553 | 0.081 | 0.312 | 0.091 | 0.144 | |
| Positive | 214 | 112(52.3) | 86(40.2) | 16(7.5) |  |  |  |  |  | |
| Negative | 139 | 80(57.6) | 55(39.6) | 4(2.9) |  |  |  |  |  | |
| Depth of invasion |  |  |  |  | 0.071 | 0.382 | 0.063 | 0.649 | 0.093 | |
| T1+T2 | 83 | 53(63.9) | 26(31.3) | 4(2.8) |  |  |  |  |  | |
| T3+T4 | 182 | 95(52.2) | 76(41.8) | 11(6.0) |  |  |  |  |  | |
| Note: ^a^, *P* was adjusted by gender, age and *H.pylori* infection status; ^b^, OR(95%CI)=0.31(0.13-0.75); ^c^, OR(95%CI)=0.31(0.14-0.70); ^d^, OR(95%CI)=0.44(0.20-0.98); ^e^, OR(95%CI)=0.64(0.42-0.97); ^f^, OR(95%CI)=0.68(0.49-0.94); ^g^, OR(95%CI)=2.69(1.22-5.92); ^h^, OR(95%CI)=1.88(1.06-3.31); ^i^, OR(95%CI)=1.56(1.07-2.27); ^j^, OR(95%CI)=1.72(1.04-2.85); ^k^, OR(95%CI)=2.50(1.10-5.66); ^l^, OR(95%CI)=2.25(1.14-4.41); ^m^, OR(95%CI)=1.45(1.00-2.11); OR, odds ratio; CI, confidence interval; *P*_corr_, *P* values after Bonferroni correction. The results are in bold if *P*<0.05. | | | | | | | | | |  |
|  |  |  |  |  |  |  |  |  |  |  |
|  |  |  |  |  |  |  |  |  |  |  |
